# Sesame cake fertilizer improves tobacco aroma quality by boosting root growth and leaf aroma precursor formation

**DOI:** 10.3389/fpls.2025.1654657

**Published:** 2025-08-20

**Authors:** Changyue Qi, Jia Lei, Weiguo Ye, Xi Zhang, Zhouwen Li, Xianyun Zhong, Dewen Tong, Shiyuan Deng, Jianjun Chen, Yuanyuan Wang

**Affiliations:** ^1^ College of Agriculture, South China Agricultural University, Guangzhou, China; ^2^ China Tobacco Guangdong Industrial Company Limited, Guangzhou, China; ^3^ Longyan Company of Fujian Provincial Tobacco Corporation, Longyan, China

**Keywords:** flue-cured tobacco, sesame cake fertilizer, root growth, leaf glandular trichomes, aromatic substances, sensory quality

## Abstract

Weak flavor in flue-cured tobacco compromises quality, making aroma enhancement crucial. Sesame cake fertilizer (SF) has the potential for improving tobacco aroma, but its effects on aroma components and mechanism remain unclear. Here, a four-year field experiment was conducted in Southern tobacco region of China to compare SF with conventional fertilization (CK). We investigated how SF influences soil quality, root and leaf development, aroma precursor accumulation, and volatile aroma composition to enhance tobacco aroma quality. Our results indicated that SF improved soil structure, pH, organic matter, and cation exchange capacity (CEC). It promoted root growth, dry matter accumulation, and root activity and CEC. Leaf photosynthesis and plastid pigment content increased due to enhanced chloroplast ultrastructure. SF also boosted glandular trichome density and secretion, leading to higher aroma precursor accumulation, particularly cembratriene-diol. After curing, 17 of 18 differential volatile aroma substances were upregulated, including carotenoids, cembratriendid alkyl degradation products, esters, terpenes, and heterocyclic compounds. Further, SF significantly increased sensory quality of flue-cured tobacco by promoting aroma quality and volume, and electronic nose analysis also confirmed this. Therefore, SF improves tobacco aroma by enhancing soil health, root growth, and leaf precursor formation. The “soil-root-leaf-differential aroma substances” framework highlights its role in increasing carotenoid and cembratriene-diol content, contributing to higher volatile aroma concentrations. This study highlights the potential of SF as a sustainable agricultural product for improving soil health and tobacco quality.

## Highlights

- Sesame cake fertilizer (SF) enhances soil quality and promote tobacco root growth.
- SF increased tobacco plastids by improving chloroplast ultrastructure in leaves.
- SF boosted aroma precursors by enhancing glandular trichome density and secretion.
- SF enriched tobacco aroma components and enhanced sensory quality.

## 1 Introduction

Tobacco (*Nicotiana tabacum* L.) is an important industrial crop worldwide, and flue-cured tobacco has the largest planting area (World Health Organization, 2017; Appau et al., 2019). Based on ecological, sensory, chemical, and metabolic characteristics, flue-cured tobacco is classified into eight types in China (Luo et al., 2019). Fujian Province, the main flue-cured tobacco producing area in southern tobacco region of China, yields high-quality fresh-sweet and honey-sweet tobacco leaves (Luo et al., 2019). However, over the past few decades, excessive chemical fertilizer use to boost yield has reduced soil quality and negatively affected crop yield and quality, including tobacco (Mulvaney et al., 2009; Zhang et al., 2018a). Soils in the Fujian tobacco-growing area are mainly acidic red and yellow soils, and prolonged excessive chemical fertilizer use has reduced organic matter, increased acidification and compaction, and imbalanced soil nutrients, resulting in uncoordinated chemical composition and weakened tobacco leaf aroma (Miao et al., 2011; Zhang et al., 2018a; Qian et al., 2019). Therefore, exploring effective fertilization application strategies to improve soil and tobacco quality is imperative.

Organic fertilizers have proven effective as soil amendments and have been widely studied in crops (Wei et al., 2016; Yang et al., 2025) such as wheat (Brar et al., 2013), maize (Feng et al., 2024), rice (Wang et al., 2024a), and tobacco (Yan and Liu, 2020). They are typically derived from animal manure, plant residues, and microbial sources rich in organic matter and beneficial microorganisms (Thuriès et al., 2002; Luo et al., 2018). Organic fertilizers improve soil structure and quality, regulate soil microbial communities, and enhance crop yield and quality (Chen et al., 2024; Yang et al., 2025). Animal manure-based organic fertilizers can improve soil structure and fertility and effectively control pests and diseases (Rowen et al., 2019). Replacing 15% of chemical nitrogen fertilizer with bioorganic fertilizer considerably improved tobacco yield and quality (Wang et al., 2024b). Combining biochar and high-carbon fertilizer with chemical fertilizers improves soil carbon and nitrogen pools, increases plant growth-promoting bacteria, enhances the aroma of tobacco leaves, and improves tobacco quality (Yan et al., 2020). Cow manure combined with various botanical oil meal organic fertilizers, including soybean, rapeseed, peanut bran, and sesame cake, improved tobacco yield and quality; however, only the combination with sesame cake substantially increased the pH of neutral tobacco soil (Chen et al., 2023). Collectively, the effects of different organic fertilizers vary in terms of soil properties and flue-cured tobacco styles.

Sesame cake fertilizer is a high-quality organic amendment that enhances tobacco quality, particularly leaf aroma (Wu et al., 2006). This effect is attributed to its rich organic matter, essential nutrients, and unique volatile components (e.g., sesamol and sesamin) that release aromatic substances during fermentation (Görgüç et al., 2019; Nouska et al., 2024). Aroma is central to tobacco style characteristics and quality, driven by specific aroma components (Banožić et al., 2020). Tobacco aroma formation is influenced by the growth environment, including soil type, fertility, and pH (Yan and Liu, 2020; Gou et al., 2024; Yan et al., 2025). Soil physicochemical conditions directly affect tobacco root growth and nutrient absorption, thereby influencing leaf development and aroma substance synthesis (Yan et al., 2020; Chen et al., 2022). Glandular trichome secretions on tobacco leaves are a primary source of aromatic substances that determine tobacco aroma after curing (Liu et al., 2022). In our previous research, we found that applying 600 kg ha^-1^ of sesame cake fertilizer effectively increased flue-cured tobacco sensory quality by improving aroma in Fujian (Qi et al., 2025). However, the mechanism underlying the increase in tobacco aroma and sensory quality after sesame cake fertilizer application remains unclear.

Therefore, a four-year fertilization trial in tobacco was conducted to (a) elucidate the effects of sesame cake fertilizer on soil physicochemical properties, tobacco growth, and flue-cured quality; and (b) systematically explain how it improves tobacco leaf quality using a “soil-root-leaf-differential aroma substances” framework.

## 2 Materials and methods

### 2.1 Site description and experimental design

The field experiment began in 2021 at Dongliu (25°20’N, 115°90’E), Fujian Province, China, with sampling primarily conducted in 2023 and 2024. This area belongs to the subtropical monsoon temperate climatic zone, with average precipitation from 151 to 236 mm and average temperature ranging from 18.3 to 20.6 °C during the tobacco growing season (**Supplementary Figure S1**). The study site soil is classified as red loam. The initial chemical characteristics of the soil were as follows: soil total nitrogen (TN) 1.13 g kg^−1^, available phosphorus (AP) 207.85 mg kg^−1^, available potassium (AK) 202.19 mg kg^−1^, organic matter (OM) 18.36 g kg^−1^, and pH 4.52. The tobacco variety used in this study was Yunyan 87, with plants transplanted in February and harvested in July.

The field experiment was conducted during the tobacco growing seasons from 2021 to 2024 using a randomized block design with two treatments: (1) control (CK), conventional fertilizer; (2) sesame cake fertilizer (SF), applied 600 kg ha^-1^ sesame cake fertilizer on top of the conventional fertilizer (**Supplementary Table S1**). The sesame cake fertilizer supplied by Zhumadian Kangbo Huixin Agricultural Science and Technology Co. China. Both CK and SF treatments received equivalent total inputs of nitrogen (150 kg N ha^-1^), phosphorus (120 kg P ha^-1^), and potassium (510 kg K ha^-1^). Organic fertilizer was applied once as the base fertilizer. Inorganic fertilizers were applied partly as a base dose (60%) and partly as topdressing (40%) across three times. Each treatment had three replicates for a total of six plots, each plot was 12 m long and 10 m wide. Ridge cultivation was conducted with a row spacing of 110 cm and a hole spacing of 45 cm. All plots received the identical irrigation, herbicide, and pesticide in accordance with local technical specifications for high-quality tobacco production.

To accurately observe changes in glandular trichomes, we conducted a pot experiment at the Qilin North Farm of South China Agricultural University, Guangzhou Province, China (23°09’N, 113°15’E) from March to June 2024. Two treatments (CK and SF) were set up with a total of 32 pots (16 pots per treatment). The tobacco variety Yunyan 87 was grown in plastic pots (39 cm diameter, 34 cm height) filled with 30 kg of clay soil from the top layer (0–40 cm) of the field, with one plant per pot. Before transplanting, the fertilizer was thoroughly mixed with the soil in each pot and incubated outdoors for seven days. The fertilizer application rate was calculated as the ratio of 1100 plants ha^-1^ in the field (**Supplementary Table S1**).

### 2.2 Sampling and measurements

#### 2.2.1 Soil properties

Soil samples were collected from a 0–40 cm depth in each plot using the five-point sampling method at the tobacco maturity stage in 2023 and 2024. Each sample was dried naturally and sieved through a 50-mesh sieve to measure the relevant indexes. Total carbon (TC) and TN were measured using a C/N elemental analyzer (Vario MAX, Elementar, Germany; Yan et al., 2020). Total phosphorus (TP) was quantified using an ascorbic acid colorimetric assay after digestion with H_2_SO_4_-HClO_4_. The total potassium (TK) was measured using a flame photometer. Available nitrogen (AN), AP, and AK were measured using alkali diffusion, molybdate-ascorbic acid, and flame atomic absorption spectroscopy, respectively. Soil pH was determined using the glass electrode method with water extraction (water-to-soil mass ratio of 2.5:1). Soil electrical conductivity (EC) was determined using a glass electrode (DDS-11A, Shanghai, China) in a 1:5 soil: water suspension (Zhang et al., 2024a). The soil organic matter (OM) was calculated using the potassium dichromate volumetric method with external heating. The soil cation exchange capacity (CEC) was determined using cobalt hexachloride hexaammonium cobalt leaching spectrophotometry (HJ 889-2017). Soil bulk density, porosity, and water content were quantified using the ring-knife, soil aggregate structure, and oven drying methods, respectively (Zou et al., 2018; Wang et al., 2024a).

#### 2.2.2 Tobacco root traits and dry matter accumulation

Intact tobacco roots were collected from three randomly sampled plants per plot at the vigorous growth (55 d after transplanting) and maturity (110 d after transplanting) stages. Each root sample was gently cleaned, and the lateral roots were separated from the main root. Roots were separately arranged and scanned with a flatbed scanner (Microtek ScanMaker i850), and root images were analyzed using WinRHIZO software to quantify the total root length (RL), surface area (RSA), and mean diameter (RMD; Jabborova et al., 2021). Root activity (RA) was determined using the triamcinolone tetrazolium chloride method (Zhao et al., 2023). Root EC and CEC were measured using a conductivity meter and the drenching method, respectively (Zhang et al., 2024a). Three plants from each plot were sampled at the same stage and dissected into roots, stems, and leaves. All separated organs were cleaned and oven-dried at 80 °C to a constant weight, and their dry weights were recorded.

#### 2.2.3 Tobacco leaf areas, function, and ultrastructure

Six plants per plot were randomly selected to measure plant height, leaf length, and leaf width at both the vigorous growth and maturity stages. Leaf area was calculated as average leaf length × average leaf width × 0.6345 (Li et al., 2024a). The net photosynthetic rate (Pn), transpiration rate (Tr), stomatal conductance (Gs), and intercellular CO_2_ molar fraction (Ci) of the middle leaves (positions 9–12 from the top) of three randomly selected plants per plot were measured between 09:00 and 11:00 am on sunny days using a Targas-1 portable photosynthesis tester (PP Systems, USA; Tor-Ngern et al., 2021).

For pigment analysis and chloroplast ultrastructure, 0.2 g of fresh leaf tissue was collected from the same vein as the central leaf, extracted with 20 mL of 95% ethanol, and incubated in the dark for 24 h. Chlorophyll a (Chla), chlorophyll b (Chlb), and carotenoid (Car) contents were determined by measuring absorbance at 665, 649, and 470 nm, respectively (Wu et al., 2014). The same position of the central leaf blade, between the 6th and 8th branch veins, was selected; tissues were cut into 1 mm × 1 mm pieces and immediately fixed in 5% glutaraldehyde solution. To observe the chloroplast ultrastructure, the fixed tissues were further treated with 1% osmium tetroxide, dehydrated in graded acetone (30–100%), and embedded in epoxy resin. Ultrathin sections were then prepared using an ultrathin microtome (Leica EM UC6, Wetzlar, Germany), double-stained with uranyl acetate and lead citrate, and observed and photographed using a transmission electron microscope (JEM-1400 Plus, Japan), with ten fields of view for each treatment (Xin et al., 2024).

#### 2.2.4 Tobacco leaf glandular trichomes and their secretion content

At the vigorous growth and maturity stages, fresh middle leaves (9th position from the top) from three randomly selected plants per treatment, avoiding the main veins, were cut and divided into two samples. One sample was immersed in a 0.2% aqueous rhodamine B solution for 20 min, rinsed three times with distilled water, and dried naturally under ventilated conditions. A 5 mm × 6 mm block of tissue was excised and observed with a super-depth-of-field microscope (VHX-500, Japan) to assess glandular trichome morphology from ten fields of view. The other sample was immediately stained with Sudan III solution, heated, dripped with dilute glycerol, and placed under a stereomicroscope (×30) to record glandular trichome density (number per millimeter) based on the average of 10 different fields of view (1.064 mm^2^ individual field area; **Supplementary Figure S2**, Zhang et al., 2018b).

Twenty leaf discs (11 cm diameter) per treatment were selected from the middle leaves to determine the leaf glandular trichome secretion content. The leaf discs were immersed in 1000 mL dichloromethane for 2 s, repeated eight times, and then 1 mL of internal standard (2.020 mg mL^-1^ sucrose octaacetate and 2.542 mg mL^-1^ heptadecan-1-ol alcohol mixture) was added. The extract was concentrated using a rotary evaporator and dried with a nitrogen blower. Gas chromatography–mass spectrometry (GC–MS) was conducted as previously described (Wang et al., 2021).

#### 2.2.5 Volatile aroma substances content of flue-cured tobacco

GC–MS analysis of volatile organic compounds (VOCs) was performed using an Agilent 7697A-8890-5977B system (Agilent, Palo Alto, CA, USA). A VF-WAXms capillary column (25 m × 0.25 mm × 0.2 µm, Agilent CP9204) was used to separate the VOCs. VOCs were extracted through headspace solid-phase microextraction (HS–SPME) as follows: Six replicates per treatment of de-stemmed and crushed flue-cured middle tobacco leaves (C3F) were collected in 20 mL headspace sample bottles. Subsequently, 2.0 µL of internal standard (50 µg/mL n-Pentadecane-d32) was added to the headspace vials. The mixture was sealed and preheated in a water bath at 80 °C for 20 min to achieve headspace equilibrium. The solid-phase microextraction (SPME) fibers were then inserted into the headspace for 20 min to adsorb VOCs. A 1 µL aliquot was injected into the GC–MS system in split mode (10:1) for analysis. The samples were separated with a VF-WAXms (25 m × 0.25 mm × 0.2 µm) capillary column with the following GC column temperature program: held at 40 °C for 2 min, increased to 100 °C at 5 °C/min, then to 230 °C at 15 °C/min and held for 5 min, followed by an additional 2 min at 230 °C. Helium (99.999%) was used as the carrier gas at a flow rate of 2 mL min^-1^. Mass spectrometry was conducted in electron impact ionization mode at 70 eV, scanning from 50 to 500 m/z at 3.2 scan/s, with the ion source maintained at 280 °C. VOCs were identified by comparing the mass spectra of all detected metabolites with those in the NIST (version 2017), MS-DIAL (version 2021), and other public databases. Data were analyzed using the Majorbio cloud platform (https://cloud.majorbio.com; Zhang et al., 2024b).

#### 2.2.6 Sensory quality and electronic nose of flue-cured tobacco

De-stemmed and shredded flue-cured middle tobacco leaves (C3F), with three replicates per treatment, were selected to evaluate the sensory quality and analyzed using an electronic nose. Sensory evaluation was performed by five experts from China Tobacco Guangdong Industrial Co., Ltd. Following the Guangdong tobacco sensory standard, evaluation indices included aroma quality (20%), aroma volume (35%), offensive taste (20%), irritancy (10%), and taste (15%), with a maximum total score of nine points. The overall odor profile was obtained using an electronic nose (PEN3, Airsense GmbH, Schwerin, Germany). Approximately 2.5 g of flue-cured tobacco sample was added to 20 mL of brown solution and equilibrated at room temperature for 1 h. Each sample was analyzed for 90 s at an injection rate of 300 mL min^-1^, and the average sensor response between 65 and 67 s was used for further analysis. The electronic nose sensor array comprised 10 metal-oxide semiconductor chemosensors to detect different odor characteristics (**Supplementary Table S2**; Zhang et al., 2024b).

### 2.3 Statistical analysis

Statistical analyses were performed using SPSS (version 26.0; SPSS Inc., Chicago, IL, USA). Significant differences were determined using student’s t-test at a 0.05 probability level. Orthogonal partial least squares discriminant analysis was performed using the SIMCA 14.1 (Umetrics, Ummea, Sweden). Different aromatic substances were screened based on variable importance in projection (VIP) > 1 and *P* < 0.05. The aroma compound descriptions were obtained from the flavor.net websites at http://www.flavornet.org and http://www.thegoodsc (Zhang et al., 2021; Wen et al., 2023). The Mantel test was performed using a Chiplot (https://www.chiplot.online), and the structural equation modeling (SEM) was constructed using the R studio “lavaan” package.

## 3 Results

### 3.1 Soil physical and chemical properties

The soil properties were considerably different after the application of SF (**Table 1**; **Supplementary Table S3**). For soil physical properties, bulk density and fractal dimension (D) under SF treatment significantly decreased by 8.20% and 6.02% on average across the year compared with CK. Water content, total porosity, proportion of large aggregates (> 0.25 mm) (R > 0.25), geometric mean diameter (GMD), and mean weight diameter (MWD) of the SF treatment on average across the year significantly increased by 22.39%, 4.63%, 8.14%, 15.17%, and 20.57%, respectively, compared with CK. For soil chemical properties, all chemical indices improved after the application of sesame cake fertilizer. Compared with CK-treated soil, the TC, TN, TK, AP, AK, OM, pH, EC, and CEC in SF-treated soil on average across the year were significantly increased by 17.23%, 10.58%, 12.20%, 15.86%, 5.43%, 19.05%, 6.39%, 22.28%, and 30.88%, respectively.

**Table 1:** Soil physical and chemical properties at tobacco maturity stage in 2023 and 2024 after applying sesame cake fertilizer.

Properties	2023		2024	
	CK	SF	CK	SF
TC (g·kg^-1^)	10.47 ± 0.07b	12.39 ± 0.14a	10.62 ± 0.18b	12.33 ± 0.05a
TN (g·kg^-1^)	1.68 ± 0.01b	1.81 ± 0.01a	1.65 ± 0.01b	1.87 ± 0.01a
TP (g·kg^-1^)	0.93 ± 0.00a	0.94 ± 0.00a	0.94 ± 0.02a	1.01 ± 0.02a
TK (g·kg^-1^)	46.54 ± 0.13b	52.15 ± 0.24a	46.84 ± 0.42b	52.61 ± 0.25a
AN (mg·kg^-1^)	139.21 ± 0.89a	143.78 ± 1.09a	141.88 ± 1.64a	146.90 ± 1.07a
AP (mg·kg^-1^)	287.47 ± 1.26b	319.79 ± 1.53a	292.65 ± 1.15b	352.34 ± 0.71a
AK (mg·kg^-1^)	221.34 ± 1.06b	232.65 ± 1.19a	230.94 ± 1.24b	244.18 ± 1.19a
рH	4.60 ± 0.01b	4.88 ± 0.01a	4.59 ± 0.09b	4.89 ± 0.01a
EC (ms·cm^-1^)	0.17 ± 0.01b	0.21 ± 0.01a	0.16 ± 0.00b	0.19 ± 0.01a
OM (g·kg^-1^)	23.68 ± 0.12b	27.55 ± 0.24a	25.12 ± 1.04b	30.55 ± 0.67a
CEC (cmol(+)·kg^-1^)	12.59 ± 0.25b	15.54 ± 0.16a	7.34 ± 0.81b	10.54 ± 0.36a
Bulk density (g·cm^-3^)	1.01 ± 0.00a	0.94 ± 0.00b	1.03 ± 0.03a	0.93 ± 0.02b
Water content (%)	0.23 ± 0.01b	0.28 ± 0.01a	0.25 ± 0.01b	0.30 ± 0.01a
Total porosity (%)	0.54 ± 0.00b	0.57 ± 0.00a	0.56 ± 0.01b	0.59 ± 0.01a
Capillary porosity (%)	0.50 ± 0.00a	0.51 ± 0.00a	0.52 ± 0.01a	0.53 ± 0.01a
Non-Capillary porosity (%)	0.05 ± 0.00a	0.06 ± 0.00a	0.04 ± 0.01a	0.06 ± 0.01a
R>0.25 mm (%)	64.13 ± 0.14b	69.63 ± 0.24a	66.14 ± 1.42b	71.26 ± 0.99a
GMD (mm)	1.16 ± 0.01b	1.26 ± 0.09a	1.21 ± 0.04b	1.43 ± 0.01a
MWD (mm)	0.53 ± 0.01b	0.67 ± 0.01a	0.58 ± 0.03b	0.71 ± 0.01a
D	2.46 ± 0.01a	2.24 ± 0.01b	2.54 ± 0.01a	2.46 ± 0.01b

CK, conventional fertilization treatment; SF, sesame cake fertilizer treatment; TC, total carbon content; TN, total nitrogen content; TP, total phosphorus content; TK, total potassium content; AN, available nitrogen content; AP, available phosphorus content; AK, available potassium content; EC, electrical conductivity; OM, organic matter; CEC, cation exchange capacity; R > 0.25mm (%), the proportion of large aggregates > 0.25mm; GMD, geometric mean diameter; MWD, mean weight diameter; D, fractal dimension. Values are presented as mean ± standard error (n=3). Different letters denote significant differences according to Student’s t-test at P < 0.05.

### 3.2 The root system traits and dry matter accumulation

At the vigorous growth stage, the year significantly affected RSA, RMD, and EC; fertilizer application significantly affected RMD and RA; while the year and fertilizer interaction only significantly affected RSA (**Table 2**). Compared with CK, the RMD significantly increased by 13.92% in 2024 after applying SF. On average across the year, RA was significantly increased by 54.32%. At the maturity stage, the year did not significantly affect any root traits; fertilizer application significantly affected RL, RMD, EC, and CEC, and the year and fertilizer interaction only significantly affected RAS. On average, across the years, RL, RSA, RMD, EC, and CEC significantly increased by 42.34%, 43.42%, 12.41%, 25.99%, and 28.01%, respectively, in the SF-treated soil compared to CK-treated soil. RA did not significantly differ between the CK and SF treatments.

**Table 2:** Tobacco root traits at the vigorous growth and maturity stage in 2023 and 2024 after applying sesame cake fertilizer.

Year	Growth stage	Treatment	Root physical traits			Root chemical traits		
			RL (cm)	RSA (cm²)	RMD (mm)	RA (ug.g.h)	EC (ms cm^-1^)	CEC (cmol (+) kg^-1^)
2023	Vigorous growth	CK	5478.19 ± 421.60a	1389.89 ± 126.29a	0.76 ± 0.01a	552.45 ± 15.03b	0.47 ± 0.03a	33.33 ± 4.41a
		SF	6330.45 ± 181.72a	1734.51 ± 78.11a	0.80 ± 0.01a	913.56 ± 83.26a	0.37 ± 0.03a	31.00 ± 3.06a
	Maturity	CK	21081.99 ± 1674.37b	5006.32 ± 481.10b	1.04 ± 0.01b	224.62 ± 18.55a	0.51 ± 0.02b	17.33 ± 0.88b
		SF	28004.47 ± 1850.68a	6768.82 ± 93.92a	1.13 ± 0.03a	302.22 ± 27.11a	0.68 ± 0.05a	22.33 ± 0.88a
2024	Vigorous growth	CK	5416.78 ± 146.94a	1874.25 ± 37.5a	0.79 ± 0.02b	516.24 ± 15.00b	0.91 ± 0.04a	24.33 ± 0.91a
		SF	6213.25 ± 734.99a	2186.28 ± 244.2a	0.90 ± 0.02a	739.64 ± 69.22a	0.89 ± 0.05a	27.17 ± 1.02a
	Maturity	CK	18157.92 ± 2079.32b	5587.98 ± 699.78b	0.99 ± 0.04b	275.90 ± 34.22a	0.59 ± 0.01b	21.12 ± 1.28b
		SF	27572.41 ± 2466.13a	8473.53 ± 613.24a	1.15 ± 0.02a	319.03 ± 20.52a	0.70 ± 0.02a	26.86 ± 1.14a
ANOVA factor	Vigorous growth	Y	ns	**	**	ns	***	ns
		F	ns	ns	**	***	ns	ns
		Y×F	ns	*	ns	ns	ns	ns
	Maturity	Y	ns	ns	ns	ns	ns	ns
		F	**	ns	**	ns	***	***
		Y×F	ns	***	ns	ns	ns	ns

CK, conventional fertilization treatment; SF, sesame cake fertilizer treatment; RL, root length; RSA, root surface area; RMD, root mean diameter; RA, root activity; EC, electrical conductivity; CEC, cation exchange capacity. Y denotes the year, F represents the fertilizer, Y×F denote their interaction effect. Values are presented as mean ± standard error (n=3). Different letters denote significant differences according to Student’s t-test at P < 0.05. ns, *, **, and *** indicate non-significance, significance at P < 0.05, < 0.01 and < 0.001 probability levels, respectively.

**Figure 1:**
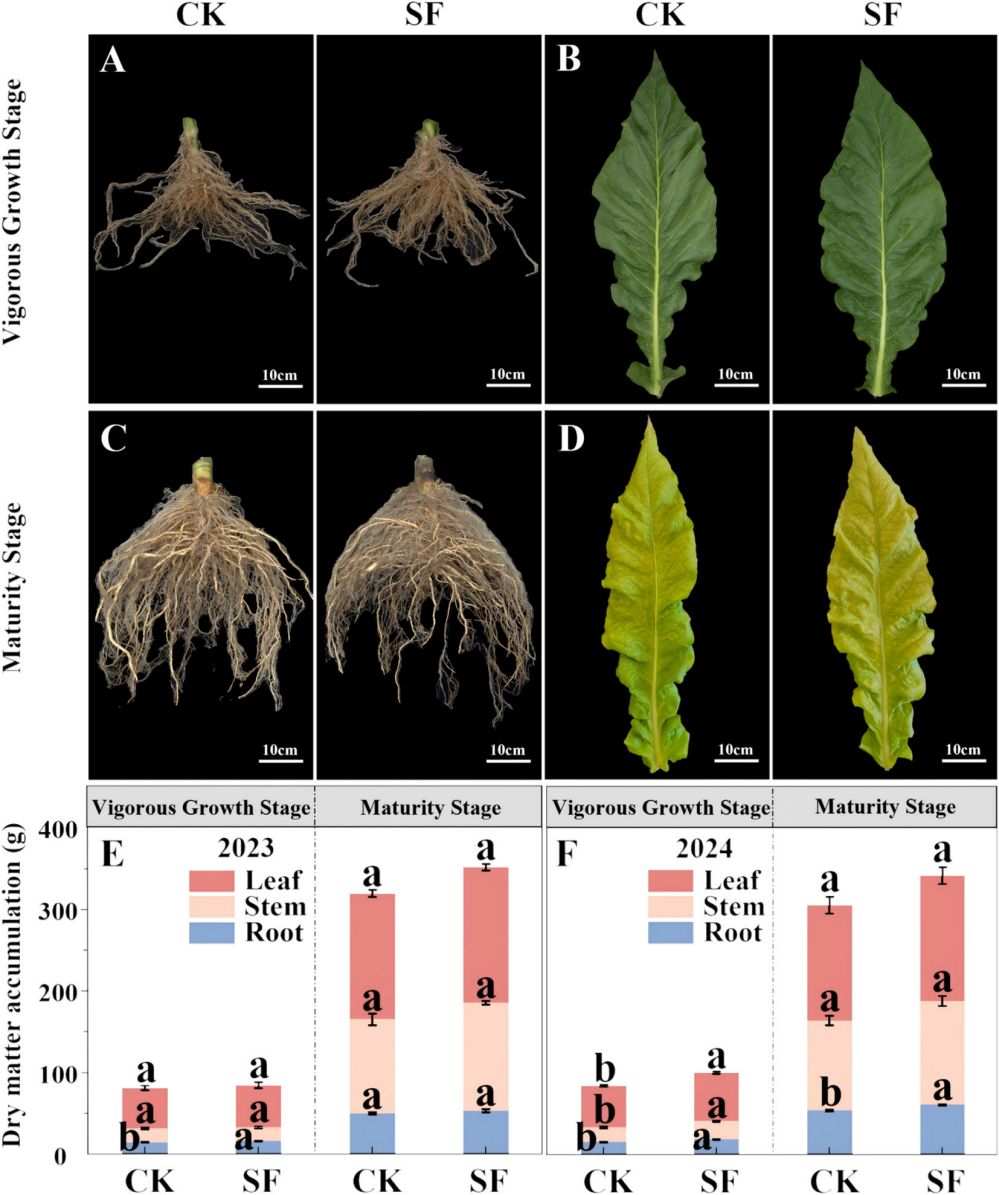
Phenograms of the tobacco root **(A, C)** and middle leaf **(B, D)**, as well as the dry matter accumulation of the leaf, stem, and root of the plant **(E, F)** at the vigorous growth and maturity stages in 2023 and 2024 after application of sesame cake fertilizer. Bars denote the SE. Different letters denote significant differences according to student’s t-test at P < 0.05, comparison within the same year and same growth stage only.

Compared with the CK, the root dry weight in the SF treatment significantly increased at the vigorous growth and maturity stages in both years compared to CK (**Figures 1A, C, E, F**). The stem and leaf dry weights did not significantly differ after SF application, except that the SF treatment was significantly higher than CK during the vigorous growth in 2024 (**Figures 1B, D–F**).

### 3.3 Leaf areas, photosynthesis, and ultrastructure

For agronomic traits, the year significantly affected plant height, leaf width, and leaf area at the vigorous growth stage and only affected leaf length at the maturity stage. Fertilizer application significantly affected leaf length, width, and area at the vigorous growth stage and only significantly affected leaf width at the maturity stage. The interaction between year and fertilizer did not significantly affect plant height or leaf area at either growth stage (**Table 3**). The plant height and leaf length were not significantly different after the application of SF, while the leaf width and leaf area were significantly increased in the SF treatment at the vigorous growth stage compared to CK but did not differ at the maturity stage.

**Table 3:** Agronomic and photosynthesis traits of tobacco leave during the vigorous growth and maturity stage in 2023 and 2024 after applying sesame cake fertilizer.

Year	Growth stage	Treatment	Agronomic traits				Photosynthesis traits			
			Height (cm)	Leaf length (cm)	Leaf width (cm)	Leaf area (cm²)	Pn (μmol CO_2_ m^-2^ s^-1^)	Gs (mol m^-2^ s^-1^)	Ci (ppm)	Tr (mmol H_2_O m^-2^ s^-1^)
2023	Vigorous growth	CK	66.33 ± 1.86a	67.33 ± 0.88a	31.83 ± 0.17b	1493.17 ± 24.06b	8.12 ± 0.34b	118.83 ± 6.32b	253.33 ± 3.12b	1.00 ± 0.04b
		SF	70.67 ± 2.33a	70.00 ± 0.58a	36.50 ± 0.29a	1667.71 ± 13.98a	11.02 ± 0.33a	228.83 ± 12.99a	294.67 ± 2.73a	1.22 ± 0.02a
	Maturity	CK	112.67 ± 0.88a	83.17 ± 2.52a	31.50 ± 1.80a	1783.87 ± 88.25a	0.86 ± 0.05b	21.33 ± 2.64a	297.0 ± 9.89a	0.31 ± 0.04a
		SF	115.00 ± 1.73a	87.67 ± 1.86a	32.50 ± 2.18a	1875.84 ± 106.07a	1.30 ± 0.08a	20.33 ± 2.06a	264.0 ± 12.16a	0.39 ± 0.05a
2024	Vigorous growth	CK	86.67 ± 1.86a	69.33 ± 1.67a	28.33 ± 1.33b	1247.85 ± 78.35b	9.80 ± 0.39b	123.20 ± 3.61b	206.67 ± 8.19b	1.39 ± 0.03b
		SF	87.33 ± 1.45a	72.33 ± 1.20a	30.67 ± 1.20a	1497.63 ± 34.34a	12.80 ± 0.24a	180.00 ± 14.25a	268.33 ± 2.30a	3.45 ± 0.04a
	Maturity	CK	112.67 ± 2.08a	89.33 ± 0.33a	31.67 ± 0.33a	1795.00 ± 22.97a	0.78 ± 0.05b	17.33 ± 3.31a	282.17 ± 19.13a	0.25 ± 0.06a
		SF	116.33 ± 0.88a	91.33 ± 0.67a	30.67 ± 0.88a	1777.87 ± 63.20a	1.29 ± 0.06a	27.33 ± 3.78a	307.33 ± 8.46a	0.37 ± 0.02a
ANOVA factor	Vigorous growth	Y	***	ns	**	**	***	ns	***	***
		F	ns	*	***	**	***	***	***	***
		Y×F	ns	ns	ns	ns	***	ns	ns	*
	Maturity	Y	ns	*	ns	ns	ns	ns	ns	ns
		F	ns	ns	***	ns	*	ns	***	ns
		Y×F	ns	ns	ns	ns	ns	ns	ns	*

CK, conventional fertilization treatment; SF, sesame cake fertilizer treatment; Pn, net photosynthetic rate; Gs, stomatal conductance; Ci, intercellular CO_2_ concentration; Tr, transpiration rate. Y denotes the year, F represents the fertilizer, Y×F denote their interaction effect. Values are presented as mean ± standard error (n=6). Different letters denote significant differences according to Student’s t-test at P < 0.05. ns, *, **, and *** indicate non-significance, significance at P < 0.05, < 0.01 and < 0.001 probability levels, respectively.

For photosynthesis traits, the year significantly affected the Pn, Ci, and Tr at the vigorous growth stage but did not affect any of the parameters at the maturity stage. Fertilizer significantly affected all parameters at the vigorous growth stage and affected both Pn and Ci at the maturity stage. The year and fertilizer interaction significantly affected both Pn and Tr at the vigorous growth stage, and only Tr at the maturity stage. Pn, Gs, Ci, and Tr were all significantly increased in the SF treatment compared to the CK treatment during vigorous growth in both years. Moreover, only Pn was significantly increased at the maturity stage in the two years; on average, across the year, the Pn in SF was 58.27% higher than in CK (**Table 3**).

**Figure 2:**
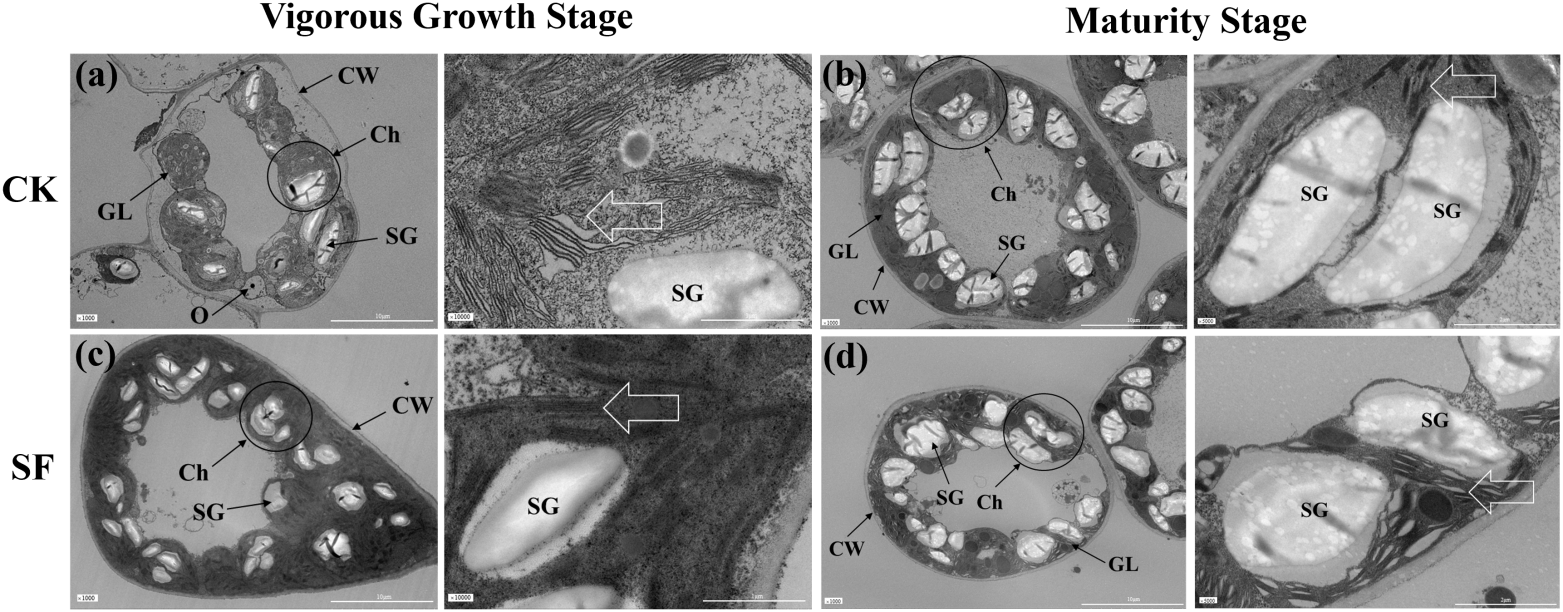
Chloroplast ultrastructure in the middle leaf of the tobacco at the vigorous growth and maturity stages in 2024 after the application of sesame cake fertilizer. Wherein, **(a, c)** represent the complete chloroplast images (scale bar = 10 μm) and lamellar structure (scale bar = 1μm) at the vigorous growth stage, respectively; **(b, d)** represent the complete chloroplast images (scale bar = 10 μm) and lamellar structure (scale bar = 2 μm) at the maturity stage, respectively. White arrows indicate chloroplast lamellar structure. CW, cell wall; GL, grana lamella; Ch, chloroplast; SG, starch granules; O, osmophilic granules.

The ultrastructure of the leaf chloroplasts was further observed; it showed an increase in chloroplast size with leaf aging, and eventually, the outer membrane ruptured to release mature starch granules (**Figure 2**). Compared to CK, at the vigorous growth stage, a higher number of chloroplasts and starch granules, more intact leaf cell walls, and thicker stacked lamellae were observed in the SF treatment. Furthermore, at the maturity stage, larger chloroplast volume, starch granule number, and laxer-stacked lamellae were observed in the SF treatment. These results indicate that tobacco leaves developed a well-organized chloroplast lamellar structure with an increased number of chloroplasts and starch granules after SF application.

### 3.4 Glandular trichomes and plastid pigment

**Figure 3:**
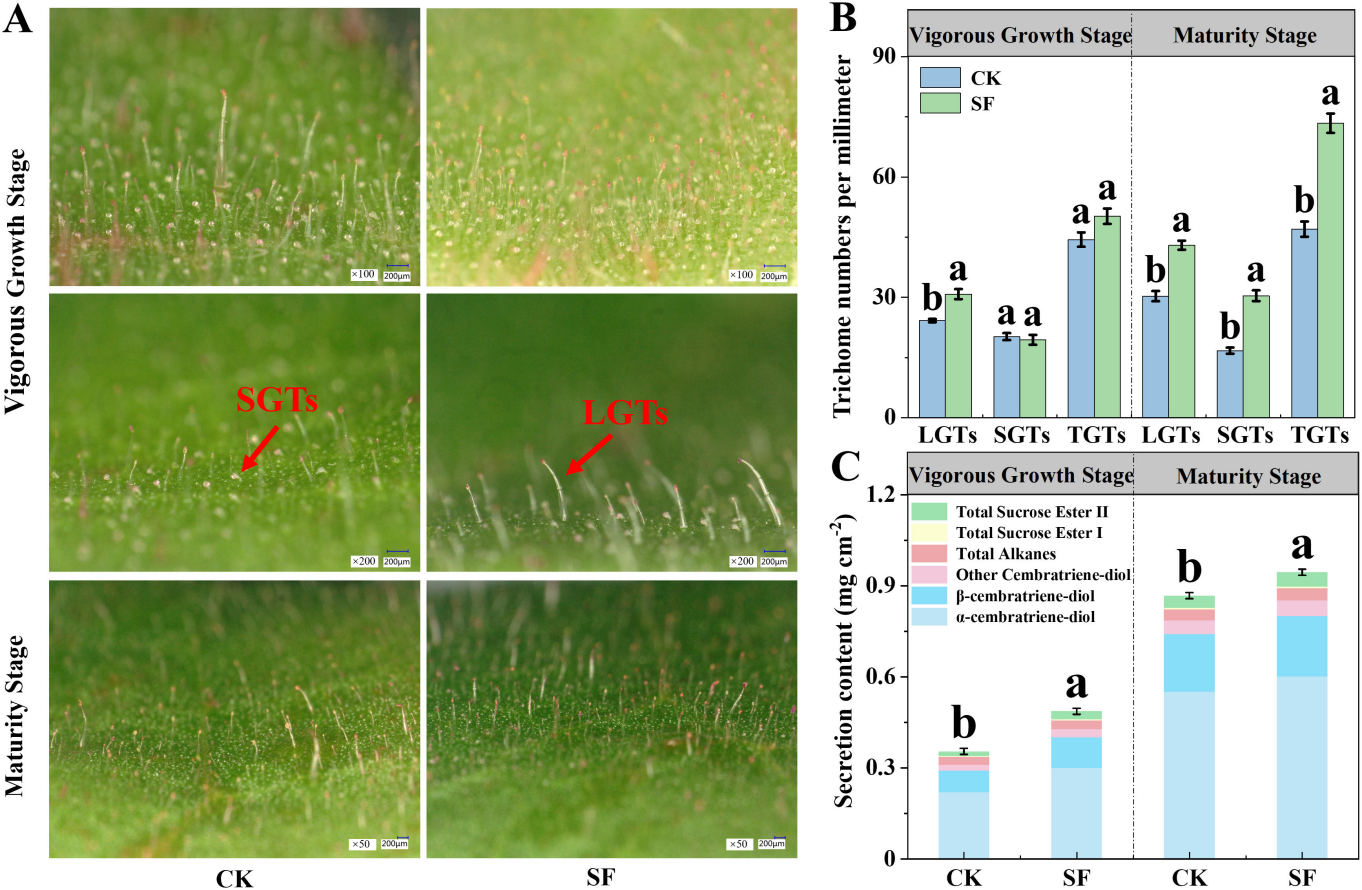
Glandular trichomes morphology **(A)**, trichome numbers per millimeter **(B)**, and trichome secretion content **(C)** in the middle-leaf in 2024 after the application of sesame cake fertilizer. Scale bar = 200 µm. LGTs, long glandular trichomes; SGTs, short glandular trichomes; TGTs, total glandular trichomes. Different letters denote significant differences according to student’s t-test at P < 0.05, comparison within the same growth stage only.

Three types of tobacco leaf glandular trichomes, long glandular trichomes (LGTs), short glandular trichomes (SGTs), and one type of non-secretory glandular trichome, exist (Meyberg et al., 1991). Aqueous rhodamine B staining of the secretory glandular trichomes (Song et al., 2022) revealed that the LGTs stain more intensely than SGTs in both treatments (**Supplementary Figure S2**). The recorded trichome numbers showed that the number of LGTs per millimeter in the SF treatment was significantly 27.27% higher than that in the CK treatment across the vigorous growth stage, and the numbers of LGTs, SGTs, and total glandular trichomes (TGTs) in the SF treatment were all significantly increased by 41.91%, 82.04%, and 56.17%, respectively, at the maturity stage compared to the CK treatment (**Figures 3A, B**; **Supplementary Figure S3**).

The total glandular trichome secretion content was significantly increased by 37.37% and 8.86% in the SF treatment at the vigorous growth and maturity stages, respectively, compared to that in the CK treatment (**Figure 3C**). At the vigorous growth stage, the secreted contents of cembratriene-diol, sucrose esters, and total alkanes in the SF treatment were significantly higher than those in the CK treatment. At the maturity stage, all secretory components were significantly higher in the SF treatment than in the CK treatment, except for total alkanes (**Supplementary Table S4**).

During the vigorous growth stage, both year and fertilizer application significantly affected the contents of chlorophyll a, b and carotenoid in tobacco leaves at the vigorous growth stage, and their interaction did not significantly affect all the plastid pigment contents (**Table 4**). The chlorophyll and carotenoid contents in the SF treatment significantly increased by 30.46% and 32.00% in 2023 and by 18.75% and 15.33% in 2024, respectively, compared to the respective CK treatment. At the maturity stage, only the year significantly affected carotenoid content.

**Table 4:** Plastid pigment content in the middle-leaf of tobacco in 2023 and 2024 after applying sesame cake fertilizer.

Year	Growth stage	Treatment	Chlorophyll a (mg L^-1^)	Chlorophyll b (mg L^-1^)	Chlorophyll (mg g^-1^)	Carotenoid (mg L^-1^)
2023	Vigorous Growth Stage	CK	10.73 ± 1.15a	4.37 ± 0.37b	1.51 ± 0.15b	1.75 ± 0.20b
		SF	13.83 ± 0.40a	5.85 ± 0.06a	1.97 ± 0.04a	2.31 ± 0.05a
	Maturity Stage	CK	3.29 ± 0.63a	1.96 ± 0.29a	0.52 ± 0.09a	0.59 ± 0.02a
		SF	3.49 ± 0.24a	2.12 ± 0.12a	0.56 ± 0.03a	0.63 ± 0.05a
2024	Vigorous Growth Stage	CK	18.32 ± 0.59b	5.63 ± 0.13b	2.40 ± 0.07b	4.11 ± 0.19b
		SF	21.48 ± 0.31a	7.04 ± 0.33a	2.85 ± 0.06a	4.74 ± 0.09a
	Maturity Stage	CK	3.27 ± 0.22a	1.58 ± 0.07a	0.49 ± 0.03a	0.96 ± 0.07a
		SF	2.17 ± 0.29b	1.80 ± 0.11a	0.40 ± 0.04a	0.84 ± 0.09a
ANOVA factor	Vigorous growth	Y	***	***	***	***
		F	**	***	***	*
		Y×F	ns	ns	ns	ns
	Maturity	Y	ns	ns	ns	***
		F	ns	ns	ns	ns
		Y×F	ns	ns	ns	ns

CK, conventional fertilization treatment; SF, sesame cake fertilizer treatment; Y denotes the year, F represents the fertilizer, Y×F denote their interaction effect. Values are presented as mean ± standard error (n=3). Different letters denote significant differences according to Student’s t-test at P < 0.05. ns, *, **, and *** indicate non-significance, significance at P < 0.05, < 0.01 and < 0.001 probability levels, respectively.

### 3.5 Volatile aroma substances in flue-cured tobacco

**Figure 4:**
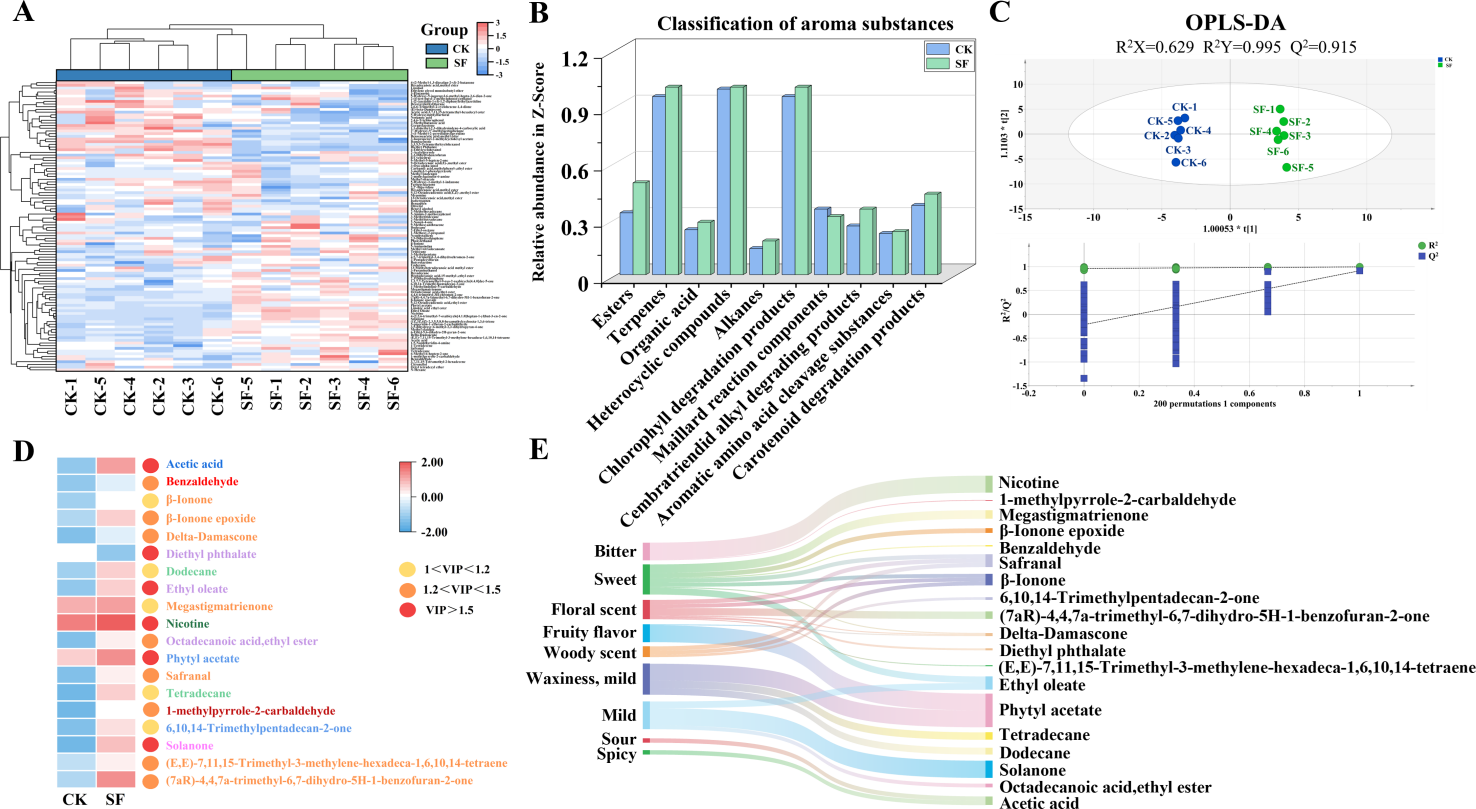
Volatile aroma substances in the middle-leaf flue-cured tobacco using HS-SPME-GC-MS in 2023 after applied sesame cake fertilizer. **(A)** Cluster heat map of volatile differential metabolites from different samples; **(B)** Aroma classification; **(C)** OPLS-DA score plot and permutation test plot; **(D)** Heat map of differential volatile substances. The words in the colors of dark blue, red, orange, light purple, light green, dark green, blue, brown, and pink represent organic acids, aromatic amino acid cleavage substances, carotenoid degradation products, esters, alkanes, heterocyclic compounds, terpenes, Maillard reaction components, and cembratriendid alkyl degrading products volatile substances, respectively; **(E)** Differential aroma classification and flavor description. Flavor descriptions on the left, differential aroma substances on the right, and the width of the middle band represent the relative content of differential aroma substances.

A total of 108 volatile substances were detected in flue-cured tobacco using HS-SPME-GC-MS (**Figure 4A**; **Supplementary Figure S4**, **Supplementary Table S5**), and 51 volatile substances with aromatic characteristics were identified (**Figure 4B**). The aroma substances were mainly divided into neutral aroma substances, including 17 carotenoid degradation products, 3 aromatic amino acid cleavage substances, 5 Maillard reaction components, 2 cembratriendid alkyl degradation products, and 1 chlorophyll degradation products, as well as other aroma substances, including 3 terpenes, 5 alkanes, 4 heterocyclic compounds, 3 organic acids, and 8 esters (**Supplementary Table S6**). Compared with CK treatment, all the aroma substances in the SF treatment were increased, except for the Maillard reaction components (**Figure 4B**).

OPLS-DA was used to analyze the effects of SF on the aromatic substances in flue-cured tobacco. The cumulative interpretation rates (R^2^X and R^2^Y) of the model were 0.629 and 0.995, respectively, and the overall predictive ability (Q2) was 0.915. The intercept between the Q2 regression curve and the Y-axis was less than zero, indicating no overfitting in this model (**Figure 4C**). The coordinates of the two treatments were clearly separated, indicating that the application of SF had a significant effect on the aroma composition of flue-cured tobacco.

Based on VIP>1 and *P*<0.05 of the first principal component in the OPLS-DA model, 19 differential aroma substances from the 51 substances were screened (**Supplementary Table S7**). The differential aromatic substances were upregulated in the SF treatment, except for diethyl phthalate (**Figure 4D**). Further, based on aroma classification and flavor description, seven differential volatile substances with the carotenoid degradation products as aroma precursors mainly provided sweet, floral, woody, and fruity flavors; one differential volatile substance with the aromatic amino acid cleavage products, Maillard reaction components, and cembratriendid alkyl degradation products as aroma precursors mainly provided sweet and mild flavors; three differential volatile substances with esters as aroma precursors mainly provided sweet, bitter, floral, and mild flavors; two differential volatile substances with terpenes and alkanes as aroma precursors mainly provided floral, woody, fruity, waxy, and mild flavors; and one differential volatile substance with the organic acid and heterocyclic compound as aroma precursors mainly provided sour and bitter flavors (**Figure 4E**; **Supplementary Table S7**; Shi and Liu, 1998; Li et al., 2024b).

### 3.6 Sensory quality and electronic nose in the flue-cured tobacco

**Figure 5:**
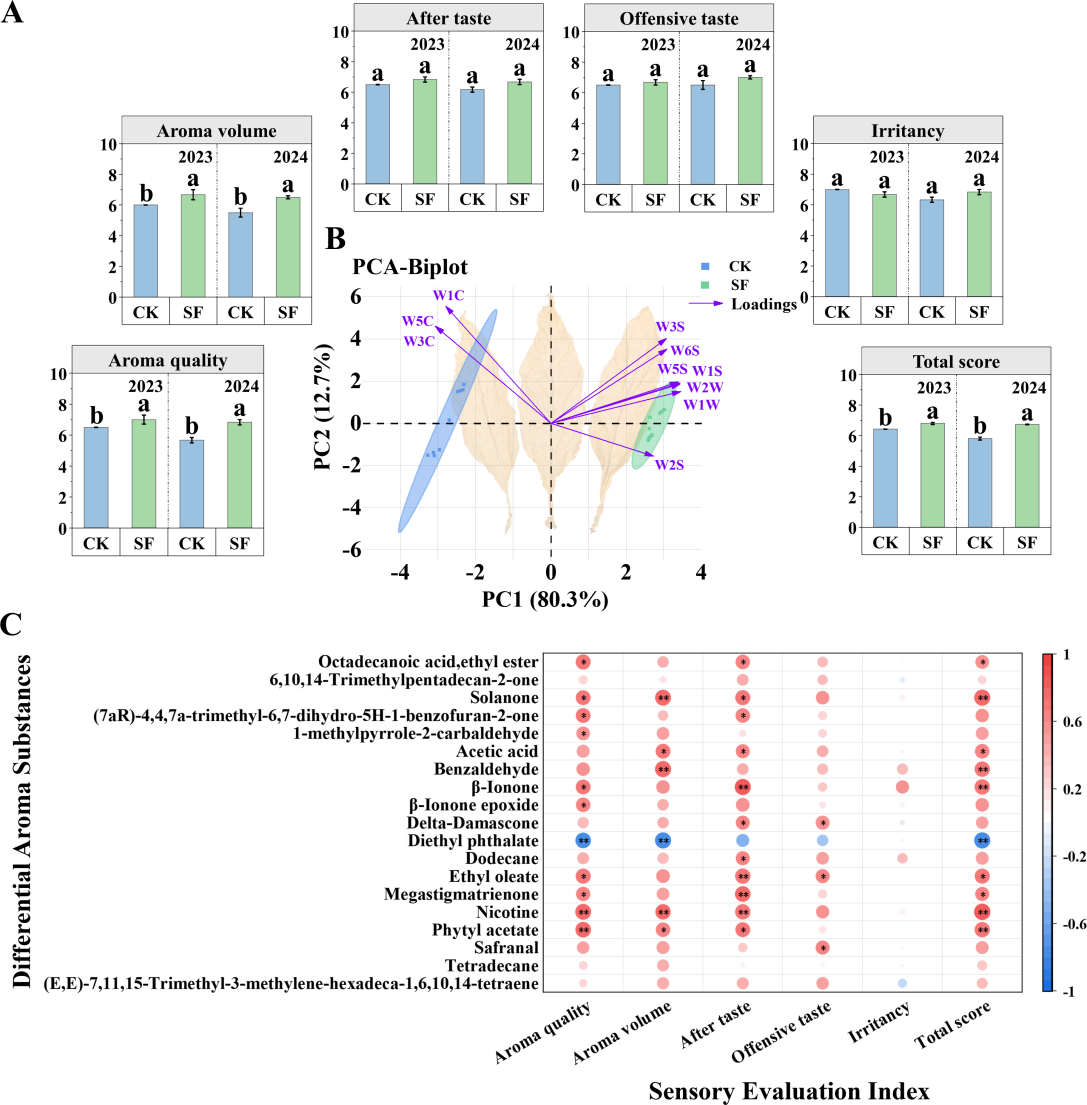
Analysis of the aroma of middle-leaf flue-cured tobacco in 2023 and 2024 after sesame cake fertilizer was applied. **(A)** Sensory evaluation of flue-cured tobacco. Total score = (aroma quality + aroma volume) × 3 + offensive taste + (irritancy + aftertaste) × 1.5; **(B)** Principal component analysis of the electronic nose and loadings of sensors; **(C)** Correlation between differential aroma substances and sensory evaluation index. Red and blue circles represent positive and negative correlations, respectively. * and ** indicate significance at P < 0.05, P < 0.01, respectively.

The total sensory quality score of flue-cured tobacco in the SF treatment was significantly higher by 5.76% and 16.03% than in the CK treatment in 2023 and 2024, respectively. This was mainly caused by the significantly increased aroma quality and volume, which were increased by 13.7% and 17.91%, respectively, in the SF treatment compared to CK on average (**Figure 5A**).

The PCA analysis of the electronic nose showed that PC1 contributed 80.3% and PC2 contributed 12.7% of the total variance (93.0%) (**Figure 5B**). The SF treatment was located in the upper right quadrant and positively correlated with W3S, W6S, W5S, W1S, W2W, and W1W, which are related to the long-chain alkanes, hydrogen, broad-range oxynitride, methane, compounds with aromatic ring structures, sulfur-containing organic compounds, sulfur, organic compounds, and terpenes. The SF treatment was also located on the negative y-axis and negatively correlated with W2S. CK was located in the upper left quadrant and was negatively correlated with W1C, W5C, and W3C, which are associated with aromatic components, hydrocarbons, and ammonia (**Supplementary Figure S5**, **Supplementary Table S2**). Therefore, the odor distributions of the SF and CK treatments were significantly different as identified by the electronic nose.

Furthermore, the relationship between sensory quality and differential aroma substances was analyzed (**Figure 5C**). Aroma quality, aroma volume, and total sensory quality score were all significantly positively correlated with solanone, nicotine, and phytyl acetate, and significantly negatively correlated with diethyl phthalate. Aftertaste was significantly positively correlated with the majority of differential aroma substances, and offensive taste was significantly positively correlated only with delta-damascone, ethyl oleate, and safranal. Irritancy was not significantly associated with differential aroma substances.

### 3.7 Relationship between tobacco growth and sensory quality with the application of sesame cake fertilizer

**Figure 6:**
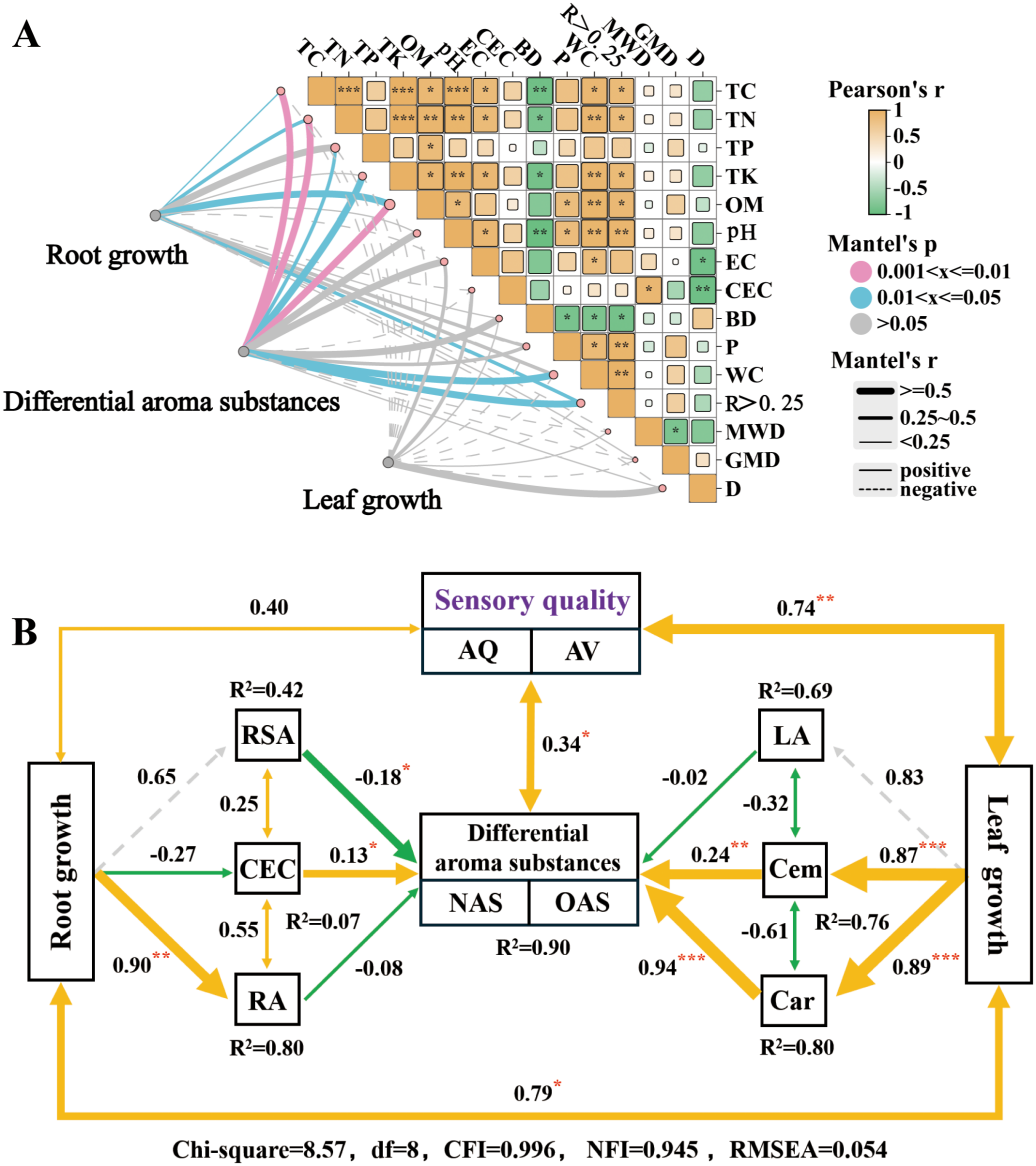
Correlation analysis **(A)** and structural equation modeling **(B)** from soil, tobacco growth, as well as sensory evaluation and aroma substances of flue-cured tobacco. **(A)** Correlation analysis between soil traits, tobacco root and leaf growth, and differential aroma substances of flue-cured tobacco after sesame cake fertilizer was applied. The thickness of the curves indicates the r-statistic using Mantel’s test, and the color denotes statistical significance. Pink and blue lines signify strong correlations at the P < 0.05 and P < 0.01 levels, respectively. Grey lines suggest no significant correlation. Solid lines indicate positive correlations, and dashed lines indicate negative correlations. The right-angled triangle represents pairwise comparisons of soil properties, with color gradients reflecting Pearson’s correlation coefficients. *, **, and *** indicate significance at P<0.05, < 0.01 and <0.001 probability levels, respectively. TC, total carbon content; TN, total nitrogen content; TP, total phosphorus content; TK, total potassium content; OM, organic matter; EC, electrical conductivity; CEC, cation exchange capacity; BD, bulk density; P, porosity; WC, water content; R > 0.25 mm (%), the proportion of large aggregates > 0.25 mm; MWD, mean weight diameter; GMD, geometric mean diameter; D, fractal dimension. **(B)** Structural Equation Modeling (SEM) illustrating the effects of root growth, leaf growth, and sensory quality based on differential aroma substances after applying sesame cake fertilizer. Solid lines indicate direct impacts, and dashed lines represent indirect impacts. Orange arrows h positive causality, and green arrows to negative causality. Numbers on the arrows and the width of the arrows represent standardized path coefficients. *, **, and *** indicate significance at P<0.05, < 0.01 and <0.001 probability levels, respectively. R^2^ indicates the explained variance score. RSA, root surface area; RA, root activity; AQ, aroma quality; AV, aroma volume; NAS, neutral aroma substances; OAS, other aroma substances; LA, leaf area; Cem, cembratriene-diol; Car, carotenoid.

Positive correlations were found between the different soil indicators, except for BD and D (**Figure 6A**). Differential aroma substances were significantly positively correlated to TC, TN, OM (p<0.01 and r≥0.5), TK, WC, R>0.25 (p<0.05 and r≥0.5), and TP (p<0.05 and 0.25<r<0.5). Root growth was significantly and positively correlated with OM (p<0.05, r≥0.5), TN and R>0.25 (p<0.05 and 0.25<r<0.5), and TC (p<0.05, r<0.25).

A SEM was constructed based on the indicators with significant correlations to further analyze the relationships (**Figure 6B**; **Supplementary Figure S6**). The model fit was assessed using the Chi-square test (χ²), with the following criteria indicating good structural equation modeling (SEM) fit: χ²/df < 3, CFI > 0.9, NFI > 0.9 and RMSEA < 0.06. Root CEC (0.13), leaf carotenoid (0.94) and cembratriene-diol (0.24) had a significant positive effect on differential aroma substances, and a further significant positive effect on sensory quality (0.34).

## 4 Discussion

### 4.1 Sesame cake fertilizer improved the soil quality and tobacco root growth

As sesame cake fertilizer (SF) was applied, the soil quality tended to increase (**Table 1**). This could be attributed to the rich nutrients in the SF, such as organic matter, proteins, and nutrient elements (Qin et al., 2024). These components synergistically improve the soil structure and enrich the nutrient pool of the soil (Vann et al., 2013; Hu et al., 2024). The significantly increased soil OM after the application of SF promoted the formation of large soil aggregates, thereby enhancing soil aggregate structure and improving total porosity (Zou et al., 2018; Sun et al., 2024; Uddin et al., 2025). Ruan et al. (2025) found that long-term organic fertilization increased the porosity by 500–1000 μm compared to inorganic fertilization. The enhanced soil aggregation and biological activities, including earthworm activity and root proliferation, further increased soil organic carbon content, which aligns with the trends observed in our study (**Table 1**). These changes reduce soil bulk density, enhance soil aeration, and increase soil water storage capacity and fertility retention (Liu et al., 2021a; Uddin et al., 2025). Improved soil aeration also promoted tobacco root growth, resulting in increased RL, RSA, and RMD, especially during the maturity stage (**Table 2**; Reichert et al., 2019). A similar response has been observed in rice (Chen et al., 2021a) and maize (Song et al., 2020), indicating that organic fertilizers can promote root growth and expansion.

The increased content of soil nitrogen, phosphorus, and potassium after the application of SF provided additional nutrients for root growth, which was also verified by the higher CEC of the soil and roots in the SF treatment (**Tables 1**, **2**; Hu et al., 2023). The higher ability to absorb and exchange nutrient ions in the soil further maintains soil fertility, and the increased root CEC and RA indicate enhanced nutrient absorption and utilization from the soil, promoting root growth and dry matter accumulation (**Figure 1**, Heintze, 1960; Singh et al., 2015; Ramos et al., 2018). Mature root systems maintain efficient material turnover even as growth decelerates, thereby sustaining functional effectiveness through optimized resource cycling (Jacobs and Timmer, 2005; Kudoyarova et al., 2011). The improvement in soil properties and effective plant growth is also derived from the increased biodiversity and interactions within microbial taxa, which boost soil metabolism (Das et al., 2022; Liu et al., 2023). Furthermore, the application of SF affects soil pH. The soil pH in Fujian Province is acidic and negatively affects tobacco growth; however, it significantly improved after the application of SF (**Table 1**; Qian et al., 2019; Wang et al., 2019). This phenomenon could be attributed to the release of base cations during the decomposition of SF, followed by displacement reactions with acidic components in the soil, effectively neutralizing the soil acidity (Eifediyi et al., 2017). Taken together, the improved soil quality after SF application established a healthy soil microenvironment that further promoted tobacco root growth and nutrient absorption and utilization.

### 4.2 Sesame cake fertilizer promotes the formation of aroma precursors in leaves

In tobacco leaves, plastid pigments are not only key to photosynthesis but also serve as essential aroma precursors, as they decompose and transform into aroma substances during the tobacco curing process (Busch and Hain, 2002; Chen et al., 2021b). The application of SF increased the chlorophyll and carotenoid content, which is consistent with previous studies on rice (**Table 4**, Liu et al., 2021b). The increased plastid pigment content could be attributed to a higher number of chloroplasts and starch granules and a well-developed chloroplast lamellar structure in tobacco leaves in the SF treatment (**Figure 2**, Zhang et al., 2024c). The increased carotenoid can degrade more volatile compounds such as β-damascone and megastigmatrienone, which imparts sweet and floral flavor to tobacco leaves, further enhancing the aroma quality and volume of cured tobacco (Shi and Liu, 1998). Our results also confirmed the promotion of the degradation pathway and increased sensory quality (**Figures 4E**, **5**). Additionally, higher plastid pigment content increases the leaf net photosynthetic rate and promotes photosynthate supply, thus increasing leaf area primarily by widening the leaf width (**Table 3**, Oguchi, 2010; Gao et al., 2023).

Tobacco aroma substances are positively correlated with glandular trichome density on the leaf, as trichome secretion serves as a critical source of aroma precursors (Liu et al., 2022). In the present study, a significant increase in both long and short glandular trichome density was observed after SF application, particularly at the maturity stage (**Figure 3B**). This may be attributed to the well-developed chloroplast structure in tobacco glandular trichomes and thylakoid granule lamella, which promote glandular trichome development during early growth stages, while sufficient photosynthate supply enhances trichome secretion accumulation (Keene and Wagner, 1985; Liang et al., 2009). Accordingly, glandular trichome secretion was also considerably increased by SF application (**Figure 3C**). The higher cembratriene-diol content not only enhanced the antimicrobial, insecticidal, and antioxidant activities of the tobacco leaves but also degraded into valuable aroma substances, mainly solanone, during the curing process, corresponding with the increased solanone content observed in the flue-cured tobacco (**Figures 3C**, **4D**; Wahlberg and Enzell, 1987).

### 4.3 Sesame cake fertilizer promoted sensory quality through increased aroma substance content in flue-cured tobacco

Aroma composition and volume are critical for the style characteristics and quality of flue-cured tobacco and are determined by the content and proportion of various aromatic substances (Geng et al., 2023). The application of SF considerably increased the sensory quality, mainly owing to a higher score in aroma quality and volume (**Figure 5**). This increase resulted from the substantially enhanced aroma substances, which is consistent with previous studies on organic fertilizer application (**Figure 4**; Yan et al., 2020; Yan and Liu, 2020). In this study, SF mainly increased compounds derived from carotenoid as precursors. Carotenoids can undergo oxidative cleavage to form terpenoid compounds such as β-Ionone, β-Ionone epoxide, and megastigmatrienone, which are primarily responsible for imparting sweet, floral, woody, and fruity flavors (Shi and Liu, 1998). These substances exhibit low odor thresholds and minimal irritation, resulting in a higher efficiency in aroma contribution compared to ordinary volatile components (Shi et al., 2023). Additionally, the increased cembratriene-diol secreted by glandular trichomes facilitated oxidative degradation to generate solanone (**Figure 3C**; **Supplementary Table S4**, Wahlberg and Enzell, 1987; Xu et al., 2024). Solanone synergizes with β-Ionone, megastigmatrienone, and other terpenoid substances, effectively balancing the harshness of smoke and enhancing the persistence and subtlety of aroma (Cai et al., 2013; Yin et al., 2019). The electronic nose analysis also reflected changes in volatile aroma substances; the sensors contributing the most were W1W (sulfurs, organic components, terpenes), W2W (aromatic ring components, organosulfur), and W5S (broad range, oxynitride; **Figure 5B**). These findings are consistent with the classification results of aroma substances detected by HS-SPME-GC-MS (**Figure 4**), wherein compounds derived from carotenoids and cembratriene-diol as precursors belonged to terpenes, while those derived from phenylalanine as precursors belonged to aromatic ring components (Cai et al., 2013; Wang et al., 2018; Liu et al., 2022). Therefore, the e-nose results verified the aroma enhancement by SF, demonstrating that it is an effective method for rapidly estimating the impact of organic fertilizer on tobacco aroma (Yang et al., 2015).

### 4.4 Comprehensive analysis of the effect of sesame cake fertilizer on tobacco quality

Based on a comprehensive analysis of soil, root, and leaf growth, and aroma substances, there was a significant relationship between soil quality, root growth, and tobacco aroma substances (**Figure 6A**). This indicates that SF in the soil enhances soil fertility and improves soil physical and chemical properties, which directly or indirectly interact with the roots, further affecting secondary metabolite production in the leaves (Singh et al., 2015; Nouska et al., 2024; Uddin et al., 2025). Subsequently, the well-developed chloroplast structure with an increased carotenoid content, as well as the higher cembratriene-diol content, accumulated more aroma substance precursors, which contributed to the increased aroma substance content in the flue-cured tobacco and ultimately improved sensory quality (**Figure 6B**; Yan et al., 2020; Liu et al., 2022; Song et al., 2022).

## 5 Conclusion

Sesame cake fertilizer application improved soil quality, increased soil and root cation exchange capacity, and promoted root growth and activity. It also enhanced leaf photosynthetic efficiency and plastid pigment content, enriched glandular trichome secretion, and increased the accumulation of key aroma precursors. Furthermore, the concentration of volatile aroma substances—including carotenoids and cembratriendid alkyl degradation products, esters, terpenes, and heterocyclic compounds—as well as the final enriched aroma quality and volume of flue-cured tobacco, were considerably enhanced. This study revealed that SF promoted plant growth and improved the aromatic quality of cured tobacco by enhancing the “soil-root-leaf-differential aroma substance” interaction, providing an effective organic fertilizer application strategy for flue-cured tobacco production.

## Data Availability

The original contributions presented in the study are included in the article/**Supplementary Material**. Further inquiries can be directed to the corresponding authors.

## Glossary

AN: Available Nitrogen
AP: Available Phosphorus
AV: Aroma Volume
BD: Bulk Density
Car: Carotenoid
Cem: Cembratriene-diol
Ch: Chloroplast
Chla: Chlorophyll a
Chlb: Chlorophyll b
CEC: Cation Exchange Capacity
Ci: Intercellular CO_2_ Concentration
CK: Conventional Fertilizer (Control)
D: Fractal Dimension
EC: Electrical Conductivity
GMD: Geometric Mean Diameter
Gs: Stomatal Conductance
HS-SPME: Headspace Solid-Phase Microextraction
LA: Leaf Area
LGTs: Long Glandular Trichomes
MS: Mass Spectrometry
MWD: Mean Weight Diameter
NAS: Neutral Aroma Substances
OM: Organic Matter
P: Porosity
Pn: Net photosynthetic rate
RA: Root Activity
RMD: Root Mean Diameter
RL: Root Length
RSA: Root Surface Area
SEM: Structural Equation Modeling
SGTs: Short Glandular Trichomes
SF: Sesame Cake Fertilizer
SPME: Solid-Phase Microextraction
TC: Total Carbon
TN: Total Nitrogen
TP: Total Phosphorus
TK: Total Potassium
Tr: Transpiration Rate
VIP: Variable Importance in Projection

## References

[B1] Appau A. Drope J. Witoelar F. Chavez J. J. Lencucha R. (2019). Why do farmers grow tobacco? A qualitative exploration of farmers perspectives in Indonesia and Philippines. Int. J. Enviro. Res. Public Health 16, 2330. doi: 10.3390/ijerph16132330, PMID: 31269640 PMC6651112

[B2] Banožić M. Jokić S. Ačkar Đ. Blažić M. Šubarić D. (2020). Carbohydrates—key players in tobacco aroma formation and quality determination. Molecules 25, 1734. doi: 10.3390/molecules25071734, PMID: 32283792 PMC7181196

[B3] Brar B. S. Singh K. Dheri G. S. Kumar B. (2013). Carbon sequestration and soil carbon pools in a rice–wheat cropping system: effect of long-term use of inorganic fertilizers and organic manure. Soil Till. Res. 128, 30–36. doi: 10.1016/j.still.2012.10.001

[B4] Busch M. Hain A. S. A. R. (2002). Functional analysis of the early steps of carotenoid biosynthesis in tobacco. Plant Physiol. 128, 439–453. doi: 10.1104/pp.128.2.439, PMID: 11842148 PMC148907

[B5] Cai K. Xiang Z. Pan W. Zhao H. Ren Z. Lei B.. (2013). Identification and quantitation of glycosidically bound aroma compounds in three tobacco types by gas chromatography–mass spectrometry. J. Chromatogr. A. 1311, 149–156. doi: 10.1016/j.chroma.2013.08.051, PMID: 24011421

[B6] Chen J. Li Y. He X. Jiao F. Zou C. (2021b). Influences of different curing methods on chemical compositions in different types of tobaccos. Ind. Crop Prod. 167, 113534. doi: 10.1016/j.indcrop.2021.113534

[B7] Chen Y. Jiang Z. Ou J. Liu F. Cai G. Tan K.. (2024). Nitrogen substitution practice improves soil quality of red soil (Ultisols) in South China by affecting soil properties and microbial community composition. Soil Till. Res. 240, 106089. doi: 10.1016/j.still.2024.106089

[B8] Chen Y. Lv X. Qin Y. Zhang D. Zhang C. Song Z.. (2023). Effects of different botanical oil meal mixed with cow manure organic fertilizers on soil microbial community and function and tobacco yield and quality. Front. Microbiol. 14. doi: 10.3389/fmicb.2023.1191059, PMID: 37303792 PMC10248155

[B9] Chen D. Wang M. Wang G. Zhou Y. Yang X. Li J.. (2022). Functional organic fertilizers can alleviate tobacco (*Nicotiana tabacum* L.) continuous cropping obstacle via ameliorating soil physicochemical properties and bacterial community structure. Front. Bioeng. Biotechnol. 10. doi: 10.3389/fbioe.2022.1023693, PMID: 36338132 PMC9631321

[B10] Chen M. Zhang S. Liu L. Wu L. Ding X. (2021a). Combined organic amendments and mineral fertilizer application increase rice yield by improving soil structure, P availability and root growth in saline-alkaline soil. Soil Till. Res. 212, 105060. doi: 10.1016/j.still.2021.105060

[B11] Das P. P. Singh K. R. Nagpure G. Mansoori A. Singh R. P. Ghazi I. A.. (2022). Plant-soil-microbes: a tripartite interaction for nutrient acquisition and better plant growth for sustainable agricultural practices. Environ. Res. 214, 113821. doi: 10.1016/j.envres.2022.113821, PMID: 35810815

[B12] Eifediyi E. K. Ahamefule H. E. Remison S. U. Aliyu T. H. Akanbi N. (2017). Effects of neem seed cake and NPK fertilizer on the growth and yield of sesame (*Sesamum indicum* L.). Cercetari Agronomice Moldova. 50, 57–72. doi: 10.1515/cerce-2017-0015

[B13] Feng W. Sánchez-Rodríguez A. R. Bilyera N. Wang J. Wang X. Han Y.. (2024). Mechanisms of biochar-based organic fertilizers enhancing maize yield on a Chinese Chernozem: Root traits, soil quality and soil microorganisms. Environ. Technol. Innov. 36, 103756. doi: 10.1016/j.eti.2024.103756

[B14] Gao Z. Li J. Z. Liu S. T. Chen Y. (2023). Within-leaf chloroplasts and nitrogen allocation to thylakoids in relation to photosynthesis during grain filling in maize. Plant Physiol.Bioch. 196, 830–840. doi: 10.1016/j.plaphy.2023.02.034, PMID: 36868131

[B15] Geng Z. He P. Gao H. Liu J. Qiu J. Cai B. (2023). Aroma precursors of cigars from different tobacco parts and origins, and their correlations with sensory characteristics. Front. Plant Sci. 14. doi: 10.3389/fpls.2023.1264739, PMID: 38192690 PMC10773810

[B16] Görgüç A. Bircan C. Mehmet F. M. (2019). Sesame bran as an unexploited by-product: Effect of enzyme and ultrasound-assisted extraction on the recovery of protein and antioxidant compounds. Food Chem. 283, 637–645. doi: 10.1016/j.foodchem.2019.01.077, PMID: 30722922

[B17] Gou Z. Zhai Z. Zhang Q. Li Y. Feng X. Zhang Y.. (2024). Characteristics of organic carbon in tobacco-growing soils with different pH. Bangladesh J. Bot. 53, 745–755. doi: 10.3329/bjb.v53i30.76614

[B18] Heintze S. G. (1960). Studies on cation-exchange capacities of roots. Plant Soil. 13, 365–383. doi: 10.1007/bf01394648

[B19] Hu W. Zhang Y. Rong X. Zhou X. Fei J. Peng J.. (2024). Biochar and organic fertilizer applications enhance soil functional microbial abundance and agroecosystem multifunctionality. Biochar 6, 3. doi: 10.1007/s42773-023-00296-w

[B20] Hu W. Zhang Y. Xiangmin R. Fei J. Peng J. Luo G. (2023). Coupling amendment of biochar and organic fertilizers increases maize yield and phosphorus uptake by regulating soil phosphatase activity and phosphorus-acquiring microbiota. Agr. Ecosyst. Enviro. 355, 11. doi: 10.1016/j.agee.2023.108582

[B21] Jabborova D. Ma H. Bellingrath-Kimura S. D. Wirth S. (2021). Impacts of biochar on basil (*Ocimum basilicum*) growth, root morphological traits, plant biochemical and physiological properties and soil enzymatic activities. Sci. Hortic. 290, 110518. doi: 10.1016/j.scienta.2021.110518

[B22] Jacobs D. F. Timmer V. R. (2005). Fertilizer-induced changes in rhizosphere electrical conductivity: relation to forest tree seedling root system growth and function. New For. 30, 147–166. doi: 10.1007/s11056-005-6572-z

[B23] Keene C. K. Wagner G. J. (1985). Direct demonstration of duvatrienediol biosynthesis in glandular heads of tobacco trichomes. Plant Physiol. 79, 1026–1032. doi: 10.1104/pp.79.4.1026, PMID: 16664523 PMC1075020

[B24] Kudoyarova G. Veselova S. Hartung W. Farhutdinov R. Veselov D. Sharipova G. (2011). Involvement of root ABA and hydraulic conductivity in the control of water relations in wheat plants exposed to increased evaporative demand. Planta 233, 87–94. doi: 10.1007/s00425-010-1286-7, PMID: 20924765

[B25] Li J. Ma Z. Dai H. Li H. Qiu J. Pang X. (2024b). Application of PLSR in correlating sensory and chemical properties of middle flue-cured tobacco leaves with honey-sweet and burnt flavour. Heliyon 10, e29547. doi: 10.1016/j.heliyon.2024.e29547, PMID: 38655300 PMC11035049

[B26] Li Y. Yang S. Dong Y. (2024a). Response of agronomic and physiological traits of flue-cured tobacco (*Nicotiana tabacum* L.) to plant stem cell soil amendments. Physiol. Mol. Plant Pathol. 131, 102290. doi: 10.1016/j.pmpp.2024.102290

[B27] Liang Z. Ji H. Zhang H. Weng M. Cui H. (2009). Morphology and structure of chloroplast in flue-cured tobacco trichomes after applying fertilizer. Acta Bot.Boreal.Occident. Sin. 29, 0291–0295. doi: 10.3321/j.issn:1000-4025.2009.02.013

[B28] Liu L. Li H. Zhu S. Gao Y. Zheng X. Xu Y. (2021b). The response of agronomic characters and rice yield to organic fertilization in subtropical China: A three-level meta-analysis. Field Crop Res. 263, 108049. doi: 10.1016/j.fcr.2020.108049

[B29] Liu X. Liu H. Zhang Y. Liu C. Liu Y. Li Z.. (2023). Organic amendments alter microbiota assembly to stimulate soil metabolism for improving soil quality in wheat-maize rotation system. J. Environ. Manage. 339, 117927. doi: 10.1016/j.jenvman.2023.117927, PMID: 37075633

[B30] Liu J. Shu A. Song W. Shi W. Gao Z. (2021a). Long-term organic fertilizer substitution increases rice yield by improving soil properties and regulating soil bacteria. Geoderma 404, 115287. doi: 10.1016/j.geoderma.2021.115287

[B31] Liu A. Yuan K. Li Q. Liu S. Li Y. Tao M.. (2022). Metabolomics and proteomics revealed the synthesis difference of aroma precursors in tobacco leaves at various growth stages. Plant Physiol. Biochem. 192, 308–319. doi: 10.1016/j.plaphy.2022.10.016, PMID: 36288661

[B32] Luo G. Li L. Friman V. P. Guo J. Guo S. Shen Q.. (2018). Organic amendments increase crop yields by improving microbe-mediated soil functioning of agroecosystems: A meta-analysis. Soil Biol. Biochem. 124, 105–115. doi: 10.1016/j.soilbio.2018.06.002

[B33] Luo D. S. Wang B. Qiao X. Y. (2019). Explanation of national regionalization of leaves style of flue-cured tobacco. Acta Tabacaria Sin. 25, 1–9. doi: 10.16472/j.Chinatobacco.2019.218

[B34] Meyberg M. Krohn S. Brümmer B. Kristen U. (1991). Ultrastructure and secretion of glandular trichomes of tobacco leaves. Flora 185, 357–363. doi: 10.1016/s0367-2530(17)30495-4

[B35] Miao Y. Stewart B. A. Zhang F. (2011). Long-term experiments for sustainable nutrient management in China. A review. Agron. Sustain. Dev. 31, 397–414. doi: 10.1051/agro/2010034

[B36] Mulvaney R. L. Khan S. A. Ellsworth T. R. (2009). Synthetic nitrogen fertilizers deplete soil nitrogen: a global dilemma for sustainable cereal production. J. Environ. Qual. 38, 2295–2314. doi: 10.2134/jeq2008.0527, PMID: 19875786

[B37] Nouska C. Deligeorgaki M. Kyrkou C. Michaelidou A. M. Moschakis T. Biliaderis C. G.. (2024). Structural and physicochemical properties of sesame cake protein isolates obtained by different extraction methods. Food Hydrocoll. 151, 109757. doi: 10.1016/j.foodhyd.2024.109757

[B38] Oguchi R. (2010). Does the photosynthetic light-acclimation need change in leaf anatomy? Plant Cell Environ. 26, 505–512. doi: 10.1046/j.1365-3040.2003.00981.x

[B39] Qi C. Zhang X. Li D. Tong D. Ye W. Li H.. (2025). Effects of equal replacement of chemical fertilisers by sesame and peanut cake fertilisers on carbon and nitrogen metabolism and nitrogen utilisation of flue-cured tobacco. Jiangsu Agric. Sci. 53, 85–93. doi: 10.15889/j.issn.1002-1302.2025.12.012

[B40] Qian X. J. Xiao J. Hong Y. F. Shi G. Q. Tan X. Zeng W. L.. (2019). Spatial and temporal variability of soil pH in Longyan tobacco planting areas. Chin. J. Trop. Crops. 40, 2061–2067. doi: 10.3969/j.issn.1000-2561.2019.10.021

[B41] Qin Z. Chang Y. L. Chen Z. M. Wang Y. G. Fan W. Gu L. B.. (2024). A novel strategy for preparing lignan-rich sesame oil from cold-pressed sesame seed cake by combining enzyme-assisted treatment and subcritical fluid extraction. Ind. Crop Prod. 218, 11. doi: 10.1016/j.indcrop.2024.119041

[B42] Ramos F. T. Dores E. F. D. C. Weber O. L. D. S. Beber D. C. Campelo J. H. Jr Maia J. C. D. S. (2018). Soil organic matter doubles the cation exchange capacity of tropical soil under no‐till farming in Brazil. J. Sci. Food. Agric. 98, 3595–3602. doi: 10.1002/jsfa.8881, PMID: 29315629

[B43] Reichert J. M. Pellegrini A. Rodrigues M. F. (2019). Tobacco growth, yield and quality affected by soil constraints on steeplands. Ind. Crop Prod. 128, 512–526. doi: 10.1016/j.indcrop.2018.11.037

[B44] Rowen E. Tooker J. F. Blubaugh C. K. (2019). Managing fertility with animal waste to promote arthropod pest suppression. Biol. Control. 134, 130–140. doi: 10.1016/j.biocontrol.2019.04.012

[B45] Ruan R. Zhang Z. Lan T. Wang Y. Li W. Chen H.. (2025). The role of soil pore structure on nitrate release from soil organic matter and applied fertilizer under three fertilization regimes. Soil Till. Res. 248, 106396. doi: 10.1016/j.still.2024.106396

[B46] Shi H. Z. Liu G. S. (1998). Tobacco aromatics (China: China Agriculture Press).

[B47] Shi J. Wu W. Zhang Y. Baldermann S. Peng Q. Wang J.. (2023). Comprehensive analysis of carotenoids constituents in purple-coloured leaves and carotenoid-derived aroma differences after processing into green, black, and white tea. Lwt 173, 114286. doi: 10.1016/j.lwt.2022.114286

[B48] Singh P. Rai R. K. Suman A. Srivastava T. K. Singh K. P. Arya N.. (2015). Soil-root interface changes in sugarcane plant and ratoon crops under subtropical conditions: implications for dry-matter accumulation. Commun. Soil Sci. Plant Anal. 46, 454–475. doi: 10.1080/00103624.2014.997385

[B49] Song X. Razavi B. S. Ludwig B. Zamanian K. Zang H. Kuzyakov Y.. (2020). Combined biochar and nitrogen application stimulates enzyme activity and root plasticity. Sci. Total Environ. 735, 139393. doi: 10.1016/j.scitotenv.2020.139393, PMID: 32492566

[B50] Song Z. Wang P. Chen X. Peng Y. Cai B. Song J.. (2022). Melatonin alleviates cadmium toxicity and abiotic stress by promoting glandular trichome development and antioxidant capacity in Nicotiana tabacum. Ecotoxicol. Environ. Saf. 236, 113437. doi: 10.1016/j.ecoenv.2022.113437, PMID: 35367878

[B51] Sun X. Niu L. Zhang M. Zhang H. Liu H. Zhao M.. (2024). Application of carbon-based nutrient fertilizer improved soil fertility and seed yield of *Paeonia ostii* ‘Feng Dan’. Ind. Crop Prod. 212, 118348. doi: 10.1016/j.indcrop.2024.118348

[B52] Thuriès L. Pansu M. Larré-Larrouy M. C. Feller C. (2002). Biochemical composition and mineralization kinetics of organic inputs in a sandy soil. Soil Biol. Biochem. 34, 239–250. doi: 10.1016/s0038-0717(01)00178-x

[B53] Tor-Ngern P. Chart-Asa C. Chanthorn W. Rodtassana C. Hasselquist N. J. (2021). Variation of leaf-level gas exchange rates and leaf functional traits of dominant trees across three successional stages in a southeast Asian tropical forest. For. Ecol. Manage. 489, 119101. doi: 10.1016/j.foreco.2021.119101

[B54] Uddin M. K. Saha B. K. Wong V. N. Patti A. F. (2025). Organo-mineral fertilizer to sustain soil health and crop yield for reducing environmental impact: a comprehensive review. Eur. J. Agron. 162, 127433. doi: 10.1016/j.eja.2024.127433

[B55] Vann M. C. Fisher L. R. Jordan D. L. David S. W. Hardy D. H. Stewart A. M. (2013). Potassium rate and application effect on flue-cured tobacco. Agron. J. 105, 304. doi: 10.2134/agronj2012.0259

[B56] Wahlberg I. Enzell C. R. (1987). Tobacco isoprenoids. Nat. Prod. Rep. 4, 237. doi: 10.1039/np9870400237, PMID: 3313125

[B57] Wang B. Deng X. Wang R. Zongguo X. Tong W. Ma E.. (2024b). Bio-organic substitution in tobacco (*Nicotiana tabacum* L) cultivation: Optimum strategy to lower carbon footprint and boost net ecosystem economic benefit. J. Environ. Manage. 370, 122654. doi: 10.1016/j.jenvman.2024.122654, PMID: 39366231

[B58] Wang S. Sun N. Zhang S. Longdoz B. Wellens J. Meersmans J.. (2024a). Soil organic carbon storage impacts on crop yields in rice-based cropping systems under different long-term fertilisation. Eur. J. Agron. 161, 127357. doi: 10.1016/j.eja.2024.127357

[B59] Wang J. Wang H. Fu Y. Huang T. Wang X. (2021). Genetic variance and transcriptional regulation modulate terpenoid biosynthesis in trichomes of nicotiana tabacum under drought. Ind. Crop Prod. 167, 113501. doi: 10.1016/j.indcrop.2021.113501

[B60] Wang H. Xu J. Liu X. Zhang D. Li L. Li W.. (2019). Effects of long-term application of organic fertilizer on improving organic matter content and retarding acidity in red soil from China. Soil Tillage Res. 195, 104382. doi: 10.1016/j.still.2019.104382

[B61] Wang J. A. Yang G. H. Li C. X. (2018). Zonal distribution of neutral aroma components in flue-cured tobacco leaves. Phytochem. Lett. 24, 125–130. doi: 10.1016/j.phytol.2018.01.014

[B62] Wei W. Yan Y. Cao J. Christie P. Zhang F. Fan M. (2016). Effects of combined application of organic amendments and fertilizers on crop yield and soil organic matter: an integrated analysis of long-term experiments. Agric. Ecosyst. Environ. 225, 86–92. doi: 10.1016/j.agee.2016.04.004

[B63] Wen S. Sun L. Zhang S. Chen Z. Chen R. Li Z.. (2023). The formation mechanism of aroma quality of green and yellow teas based on GC-MS/MS metabolomics. Food Res. Int. 172, 113137. doi: 10.1016/j.foodres.2023.113137, PMID: 37689901

[B64] World Health Organization (WHO) (2017). Tobacco and its environmental impact: an overview. Geneva: World Health Organization. Available online at: http://apps.who.int/iris.

[B65] Wu Q. Wu X. Zhang X. Jiang C. Xiao B. Zhang Y.. (2014). Mapping of two white stem genes in tetraploid common tobacco (*Nicotiana tabacum* L.). Mol. Breed. 34, 1065–1074. doi: 10.1007/s11032-014-0097-0

[B66] Wu X. P. Zhong X. M. Qin Y. Q. Liu S. (2006). Effects of proportional application of sesame seed cake fertilizers and chemical fertilizers on the aroma quality of flue-cured tobacco leaves. Acta Agronomica Sinica. 32, 1554–1559. doi: 10.3321/j.issn:0496-3490.2006.10.021

[B67] Xin L. Tang M. Zhang L. Huang W. Wang X. Gao Y. (2024). Effects of saline-fresh water rotation irrigation on photosynthetic characteristics and leaf ultrastructure of tomato plants in a greenhouse. Agric. Water Manage. 292, 108671. doi: 10.1016/j.agwat.2024.108671

[B68] Xu M. Du Y. Hou X. Zhang Z. Yan N. (2024). Chemical structures, biosynthesis, bioactivities, and utilisation values for the diterpenes produced in tobacco trichomes. Phytochemistry 223, 114117. doi: 10.1016/j.phytochem.2024.114117, PMID: 38697243

[B69] Yan S. Liu G. (2020). Effect of increasing soil carbon content on tobacco aroma and soil microorganisms. Phytochem. Lett. 36, 42–48. doi: 10.1016/j.phytol.2020.01.011

[B70] Yan S. Wang P. Cai X. Wang C. Zwieten L. V. Wang H.. (2025). Biochar-based fertilizer enhanced tobacco yield and quality by improving soil quality and soil microbial community. Environ. Technol. Innov. 37, 103964. doi: 10.1016/j.eti.2024.103964

[B71] Yan S. Zhao J. Ren T. Liu G. (2020). Correlation between soil microbial communities and tobacco aroma in the presence of different fertilizers. Ind. Crop Prod. 151, 112454. doi: 10.1016/j.indcrop.2020.112454

[B72] Yang J. An H. Li J. Zheng J. Long J. (2015). Study on analysis of volatile components in cigarette made through different drying processing by electronic nose. Chem. Res. Application. 27, 246–250. doi: 10.3969/j.issn.1004-1656.2015.03.002

[B73] Yang J. Ren Y. Jia M. Huang S. Guo T. Liu B.. (2025). Improving soil quality and crop yield of fluvo-aquic soils through long-term organic-inorganic fertilizer combination: promoting microbial community optimization and nutrient utilization. Environ. Technol. Innov. 37, 104050. doi: 10.1016/j.eti.2025.104050

[B74] Yin F. Karangwa E. Song S. Duhoranimana E. Lin S. Cui H.. (2019). Contribution of tobacco composition compounds to characteristic aroma of Chinese faint-scent cigarettes through chromatography analysis and partial least squares regression. J. Chromatogr. B. 1105, 217–227. doi: 10.1016/j.jchromb.2018.12.001, PMID: 30611933

[B75] Zhang W. Cao J. Li Z. Li Q. Lai X. Sun L.. (2021). HS-SPME and GC/MS volatile component analysis of yinghong no. 9 dark tea during the pile fermentation process. Food Chem. 357, 129654. doi: 10.1016/j.foodchem.2021.129654, PMID: 33866239

[B76] Zhang M. Guo D. Wu G. Han P. Shi Y. Zheng T.. (2024b). Analysis of volatile compound metabolic profiles during the fermentation of filler tobacco leaves through integrated E-nose, GC–MS, GC-IMS, and sensory evaluation. J. Chromatogr. A. 1737, 465472. doi: 10.1016/j.chroma.2024.465472, PMID: 39467511

[B77] Zhang K. Han X. Fu Y. Khan Z. Zhang B. Bi J.. (2024c). Biochar coating promoted rice growth under drought stress through modulating photosynthetic apparatus, chloroplast ultrastructure, stomatal traits and ROS homeostasis. Plant Physiol. Bioch. 216, 109145. doi: 10.1016/j.plaphy.2024.109145, PMID: 39321623

[B78] Zhang S. T. Song X. N. Li N. Zhang K. Liu G. S. Li X. D.. (2018a). Influence of high-carbon basal fertiliser on the structure and composition of a soil microbial community under tobacco cultivation. Res. Microbiol. 169, 115–126. doi: 10.1016/j.resmic.2017.10.004, PMID: 29122672

[B79] Zhang B. Zhang Z. Bai X. Li L. Wu J. Liu Y.. (2024a). Long-term rice cultivation enhances root development and yields by improving the structural properties of soil aggregates in saline–alkaline environments. Environ. Technol. Innov. 36, 103848. doi: 10.1016/j.eti.2024.103848

[B80] Zhang H. Y. Zhang S. T. Yang Y. X. Jia H. F. Cui H. (2018b). Metabolic flux engineering of cembratrienol production in both the glandular trichome and leaf mesophyll in nicotiana tabacum. Plant Cell Physiol. 3, 566–574. doi: 10.1093/pcp/pcy004, PMID: 29346685

[B81] Zhao S. Wang W. Chen X. Gao Y. Wu X. Ding M.. (2023). Graphene oxide affected root growth, anatomy, and nutrient uptake in alfalfa. Ecotoxicol. Environ. Saf. 250, 114483. doi: 10.1016/j.ecoenv.2022.114483, PMID: 36586166

[B82] Zou C. M. Li Y. Huang W. Zhao G. K. Pu G.r. Su J. E.. (2018). Rotation and manure amendment increase soil macro-aggregates and associated carbon and nitrogen stocks in flue-cured tobacco production. Geoderma 325, 49–58. doi: 10.1016/j.geoderma.2018.03.017

