# Supplementary material for: Sesame cake fertilizer improves tobacco aroma quality by boosting root growth and leaf aroma precursor formation
Source: Front Plant Sci. 2025 Aug 20;16:1654657. doi: 10.3389/fpls.2025.1654657 (PMC12406223; doi:10.3389/fpls.2025.1654657)
Supplement: Supplementary file 2 [file DataSheet1.docx]

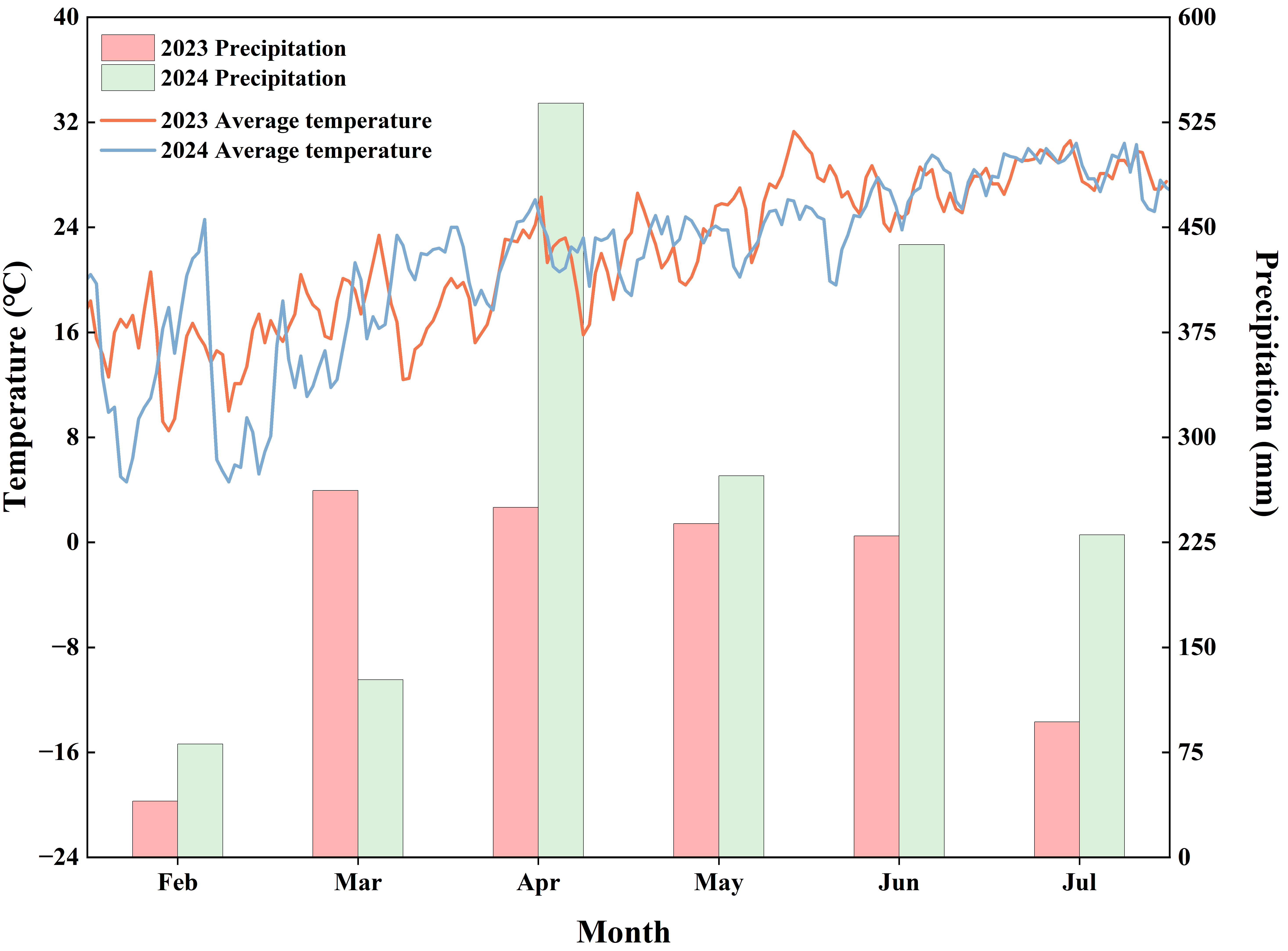


**Supplementary Fig. 1** Monthly mean precipitation and daily mean temperature during the tobacco growing season from February to July in 2023 and 2024.


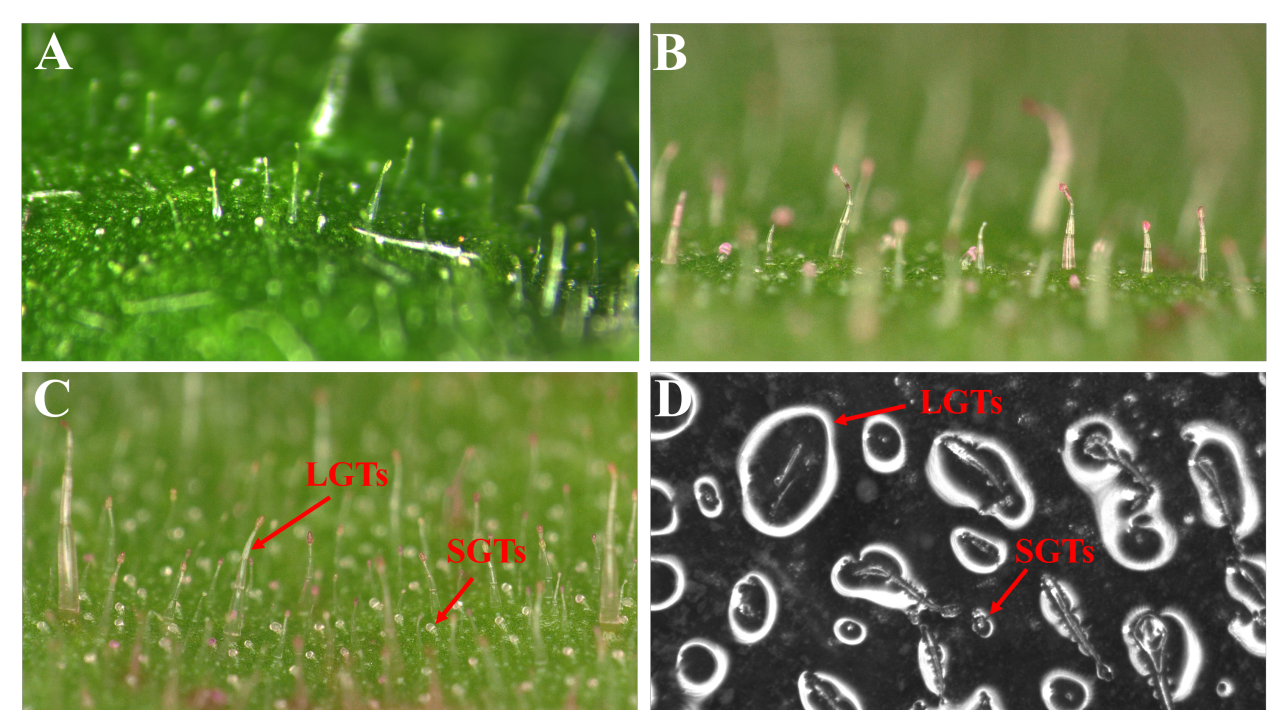


**Supplementary Fig. 2** Types of glandular trichomes in tobacco. (A) Unstained glandular trichomes (×50); (B) Rhodamine B stained glandular trichomes (×200); (C) Morphological observation of glandular trichomes. LGTs, long glandular trichomes; SGTs, short glandular trichomes (×100); (D) Glandular trichomes stained with Sudan III, large apertures are the LGTs and small apertures are the SGTs (×80).


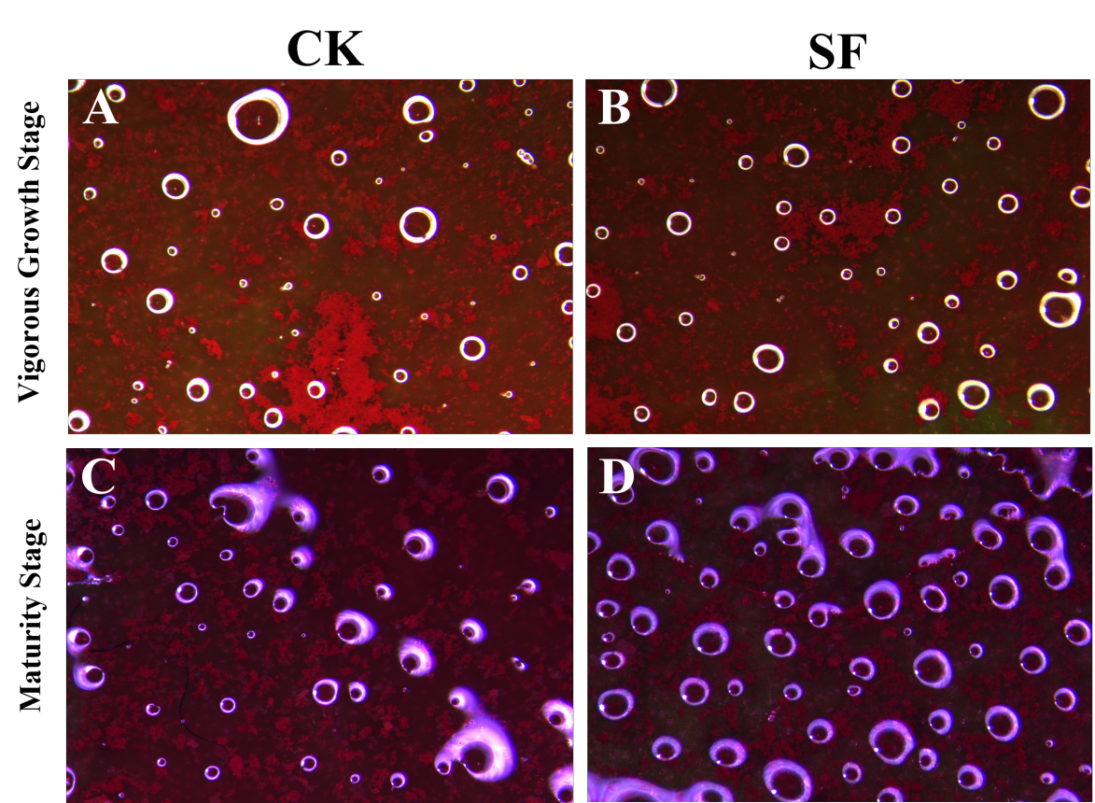


**Supplementary Fig. 3** Comparison of the trichome numbers per millimeter in the middle-leaf at the vigorous growth (A, B) and maturity stages (C, D) in 2024 after applying sesame cake fertilizer. The large apertures were the LGTs, and small apertures were the SGTs (×30).


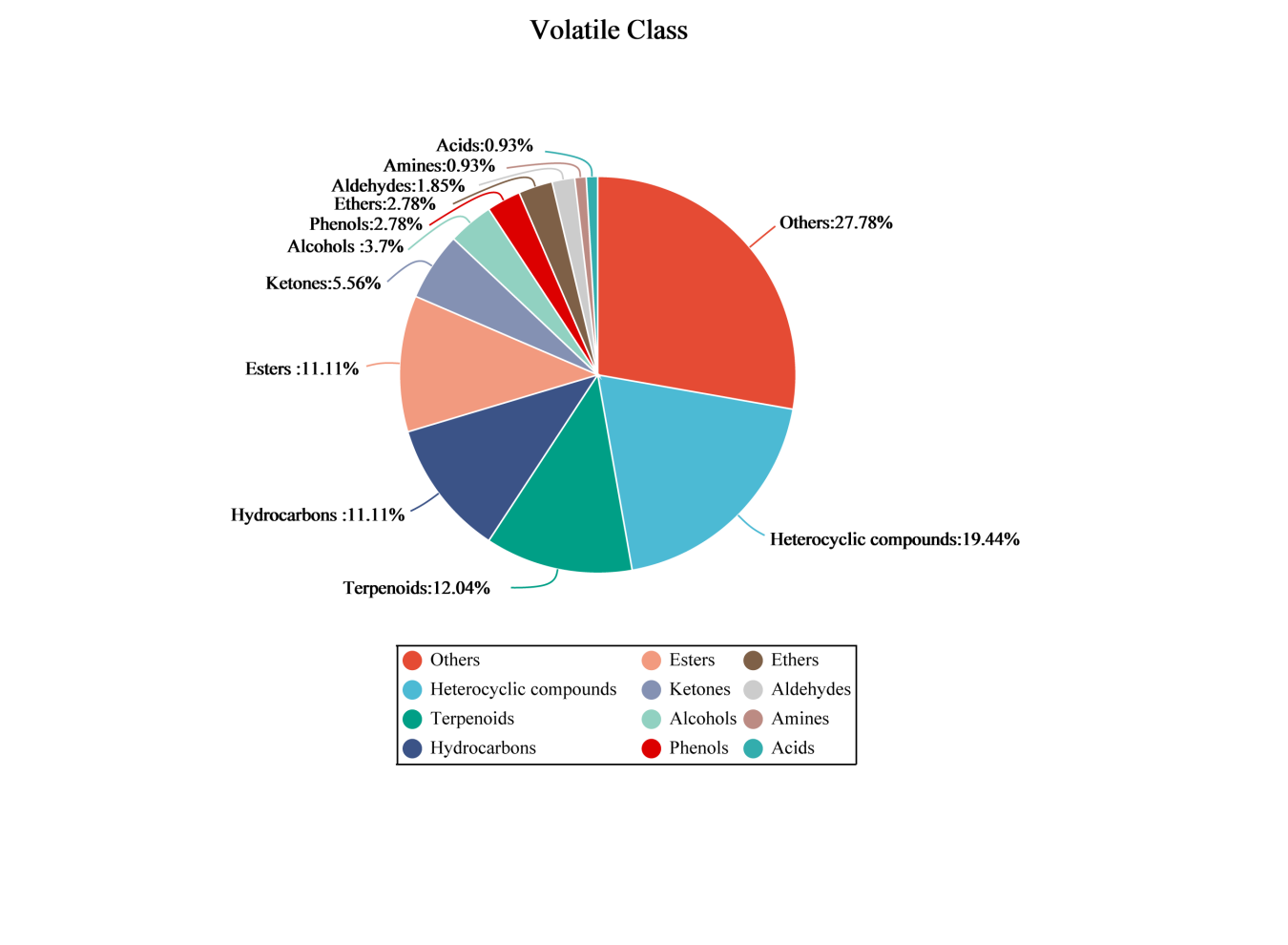


**Supplementary Fig. 4** Classification of 108 volatile substances in the middle-leaf of tobacco identified by HS-SPME-GC-MS.


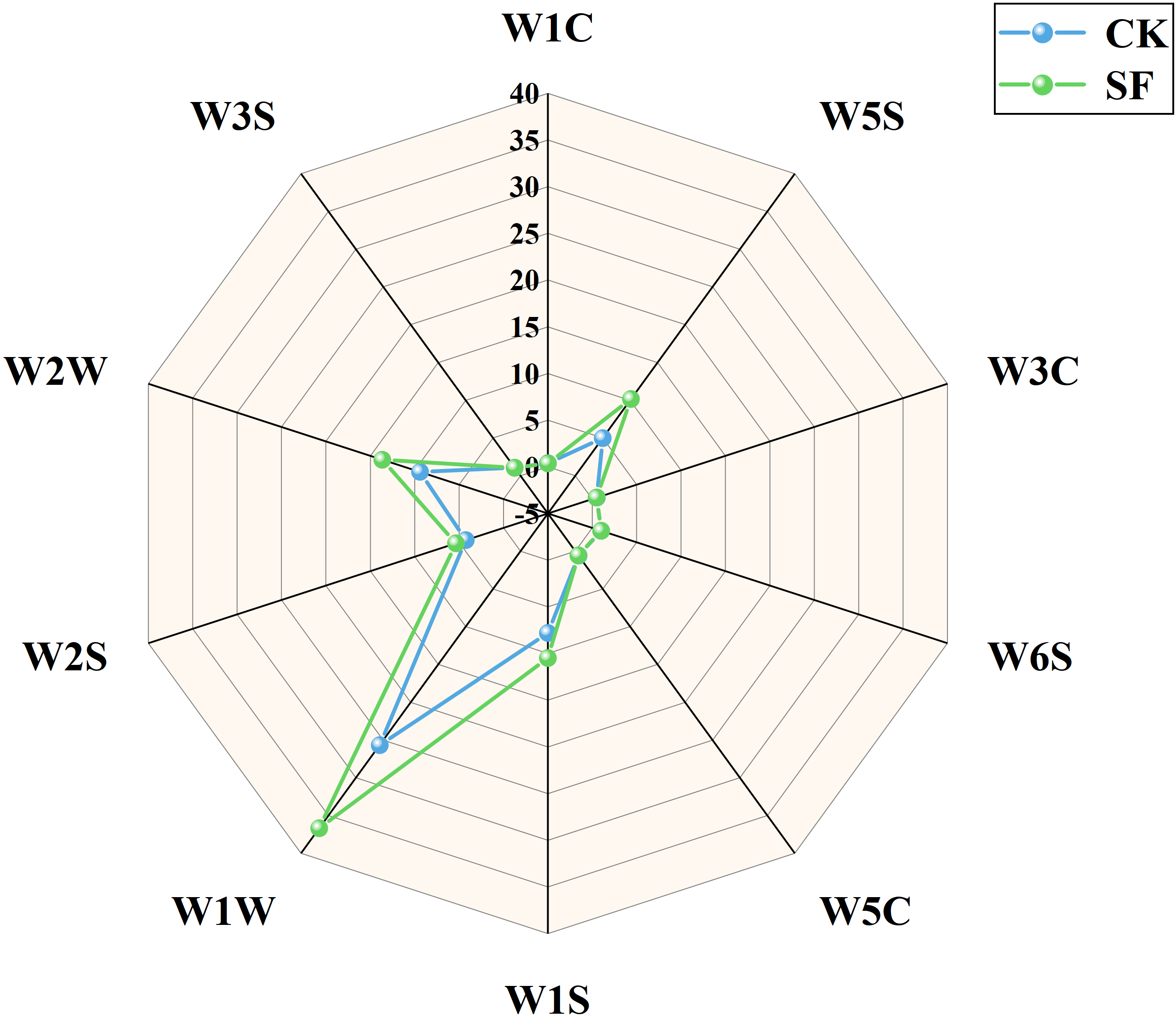


**Supplementary Fig. 5** Aroma profile in the middle-leaf of flue-cured tobacco in 2023 using an electronic nose after applying sesame cake fertilizer.


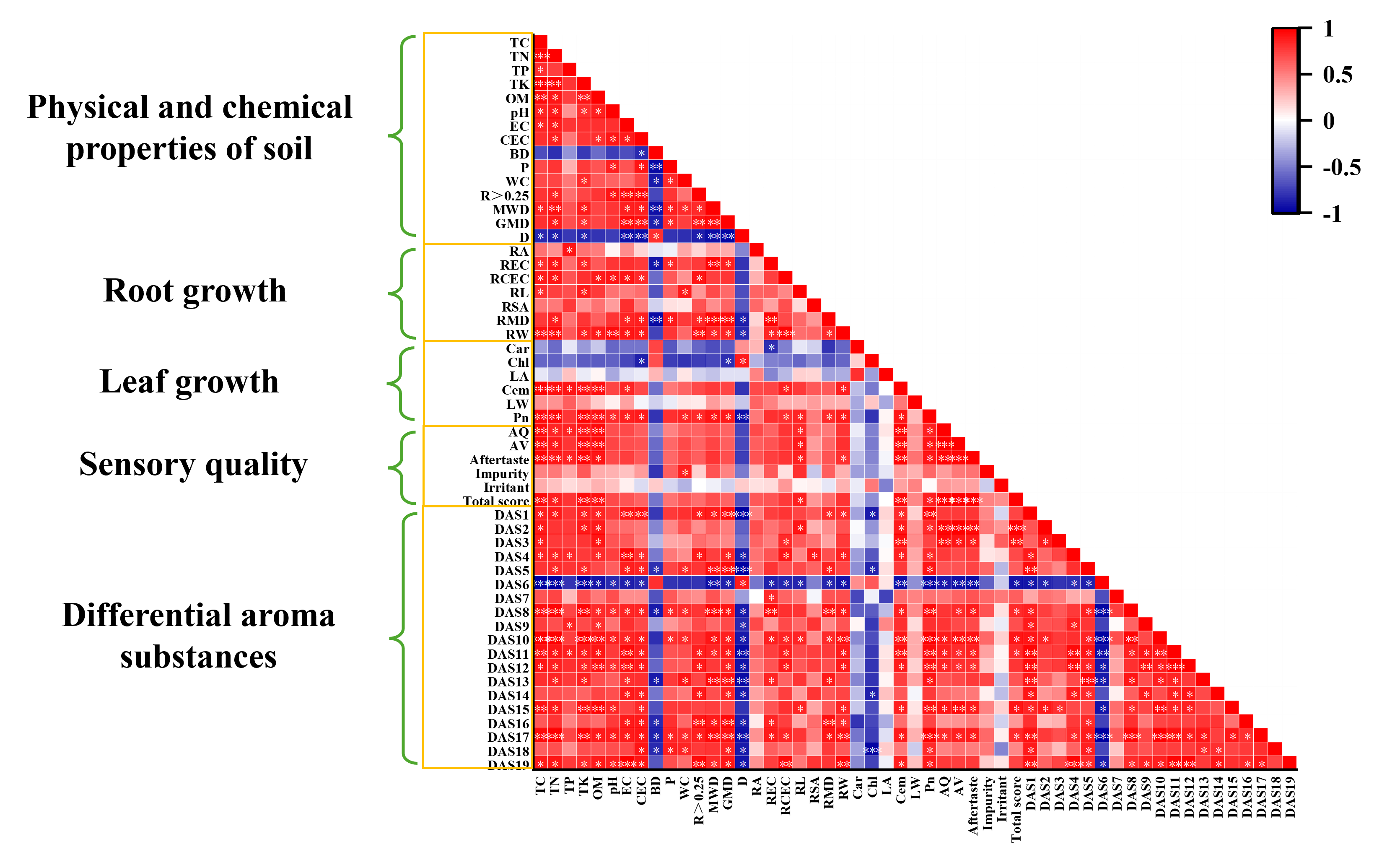


**Supplementary Fig. 6** Correlation analysis among soil physico-chemical properties, tobacco root growth, leaf growth, and differential aroma substances after applying sesame cake fertilizer. TC, total carbon content; TN, total nitrogen content; TP, total phosphorus content; TK, total potassium content; OM, organic matter; EC, electrical conductivity; CEC, cation exchange capacity; BD, Bulk Density; P, Porosity; WC, Water Content; R>0.25mm (%), the proportion of large aggregates >0.25mm; GMD, geometric mean diameter; MWD, mean weight diameter; D, fractal dimension. RA, root activity; REC, root electrical conductivity; RCEC, root cation exchange capacity; RL, root length; RAS, root surface area; RMD, root mean diameter; RW, root weight; Car, Carotenoid; LA, leaf area; Cem, Cembratriene-diol; LW, leaf width; Pn, net photosynthetic rate; AQ, aroma quality; AV, aroma volume; DAS1, acetic acid; DAS2, benzaldehyde; DAS3, β-Ionone; DAS4, β-Ionone epoxide; DAS5, Delta-Damascone; DAS6, Diethyl phthalate; DAS7, dodecane; DAS8, ethyl oleate; DAS9, megastigmatrienone; DAS10, nicotine; DAS11, octadecanoic acid, ethyl ester; DAS12, phytyl acetate; DAS13, safranal; DAS14, tetradecane; DAS15, 1-methylpyrrole-2-carbaldehyde; DAS16, 6,10,14-Trimethylpentadecan-2-one; DAS17, solanone; DAS18, (E,E)-7,11,15-Trimethyl-3-methylene-hexadeca

-1,6,10,14-tetraene; DAS19, (7aR)-4,4,7a-trimethyl-6,7-dihydro-5H-1-benzofuran

-2-one. *, **, and *** indicate significance at P < 0.05, < 0.01 and < 0.001 probability levels, respectively.
